# Ecological Processes Underpinning Microbial Variability in Archean Granitoids Beneath the Deccan Traps: Evidence From Deep Drilling in Koyna, India

**DOI:** 10.1111/1758-2229.70351

**Published:** 2026-05-03

**Authors:** Rajendra Prasad Sahu, Sufia Khannam Kazy, Debarshi Mukherjee, Sukanta Roy, Thomas Wiersberg, Pinaki Sar

**Affiliations:** ^1^ Environmental Microbiology and Genomics Laboratory, Department of Bioscience and Biotechnology Indian Institute of Technology Kharagpur Kharagpur West Bengal India; ^2^ Department of Biotechnology National Institute of Technology Durgapur Durgapur West Bengal India; ^3^ Ministry of Earth Sciences, Borehole Geophysics Research Laboratory Karad India; ^4^ GFZ Helmholtz Centre for Geosciences Potsdam Germany

**Keywords:** Archean granitic basement, deep biosphere, ecological processes, environmental drivers, Koyna Seismic Zone

## Abstract

The deep continental rocky biosphere harbours a diverse and active microbial ecosystem which mostly remains elusive. Here we investigate the ecological processes shaping the microbial species distribution and community assembly across the environmental gradients within the extreme realm of Archean granitic basement (down to ~3000 mbs) of the Koyna Seismic Zone (KSZ), Deccan Traps province, India. Amplicon‐based metataxonomy reveals a predominance of bacteria with a few dominant taxa present across the depth, while a large number of rare taxa (< 0.01% abundance) remain localized. Multivariate analyses suggest a distinct partitioning of the KSZ microbial ecosystem into shallow, intermediate, and deeper zones, with a pronounced effect of depth, Fe_2_O_3_, TOC, and NO_2_^−^ on community variability. Phylogenetic null models delineate high turnover among the microbial communities, highlighting the major role of dispersal limitation and variable selection in community assembly. Co‐occurrence analysis shows high positive correlation indicating mutualistic association for efficient nutrient utilization and minimization of diversity loss. The potential influence of seismic activity on the microbial community assemblages in specific regions is inferred from correlating cohesion patterns with ^4^He and acetate concentrations. Overall results indicate that microbial communities within the granitoid basement of KSZ are shaped by variable environmental conditions, limited dispersion, and mutualistic interactions between them.

## Graphical abstract

Deep biosphere hosted by Archean granitoid basement of Deccan Traps showed depth‐wise microbial partitioning. Limited dispersion and variable selection control community assembly. Fewer abundant bacterial taxa were ubiquitous, while large numbers of rare taxa remained localized. Cooperative interactions facilitate optimal nutrient utilization by the microorganisms with minimum diversity loss.

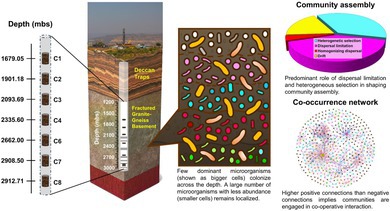

## 1 Introduction

Terrestrial deep biosphere extending down to several kilometres below the surface, within the extreme realm of crystalline granitic crust represents one of the largest biomes of our planet (Beaver and Neufeld 2024; Templeton and Caro 2023; Kieft 2016). Understanding the ecology, adaptation and evolution of myriad microorganisms inhabiting the extreme and resource‐poor confines of crystalline rock remains limited and represents new research frontiers (Beaver and Neufeld 2024; Templeton and Caro 2023; Amils et al. 2023; Acciardo et al. 2025). Gaining insights into these ecosystems is highly imperative for ascertaining their biogeochemical role, and answering fundamental questions related to the origin and evolution of life on Earth and possibly beyond (Colman et al. 2017; Magnabosco et al. 2018). Recent studies highlight the importance of deep biosphere communities in the sustainability of industries that rely on deep biosphere, for example, hydrocarbon industry, geo‐sequestration of CO_2_ and deep geological disposal of hazardous wastes (Herzig et al. 2023). Studies conducted during the past few decades provide valuable insights into the diversity and function of terrestrial deep biosphere microorganisms through investigation of groundwater, but are yet unable to directly relate to the pertinent ecological processes that drive the community, especially those hosted by intrinsically complex crystalline rock matrix with diverse physical and chemical gradients. Despite the recent scientific progress (see, for example, Amils et al. (2023), Dutta et al. (2018), Soares et al. (2023), Purkamo et al. (2020), Dai et al. (2021)), details of the ecological processes underpinning the continental deep crust hosted microbial ecosystems including their geochemical drivers, assembly processes, dynamics, turnover, interconnections and complexity are yet to be fully understood. Unravelling these processes is considered key to comprehend the foundational principles of the microbial ecology of this extraordinary biome.

There is a growing understanding of the terrestrial deep biosphere mostly with aquatic (ground‐, fault‐, fracture‐ water) and a few crystalline bedrock‐hosted microbial communities across the continents (reviewed by Beaver and Neufeld (2024), Templeton and Caro (2023), Acciardo et al. (2025), Magnabosco et al. (2018), Kazy et al. (2021), Escudero and Amils (2023), Amano et al. (2024), González‐Rosales et al. (2025)). Most studies have brought forth the diversity, abundance, and composition of the microbial communities across varying lithology and sampling depths, with a few emphasizing their biogeochemical or geobiological attributes (Amils et al. 2023; Purkamo et al. 2020; Dai et al. 2021; Quraish et al. 2024; Nyyssönen et al. 2014). A few studies have also highlighted the key roles of temperature, H_2_, rock porosity, mineralogy, and hydrological connectivity in influencing microbial communities in various crystalline habitats (Magnabosco et al. 2018; Dai et al. 2021; Zhang et al. 2022; Itävaara et al. 2011). However, studies aimed at understanding the fundamental ecological processes driving microbial communities under the extreme and resource‐limited deep crust‐hosted biosphere are very limited. With respect to the ecological processes governing microbial assembly within the deep subsurface, the interplay of both stochastic and deterministic processes has been suggested (Templeton and Caro 2023). Recent studies on subsurface aquifers have shown a greater role of stochastic processes, especially dispersal limitation and fluid advection, along with variable selection (a type of deterministic process) in shaping community assembly (Zhang et al. 2022; Putman et al. 2021). However, the delineation of such processes within the deep crystalline continental crust, kilometers below the surface, has not been done satisfactorily, mostly due to the limited accessibility of such habitats and the prohibitive cost of drilling (Kieft 2022).

**FIGURE 1:**
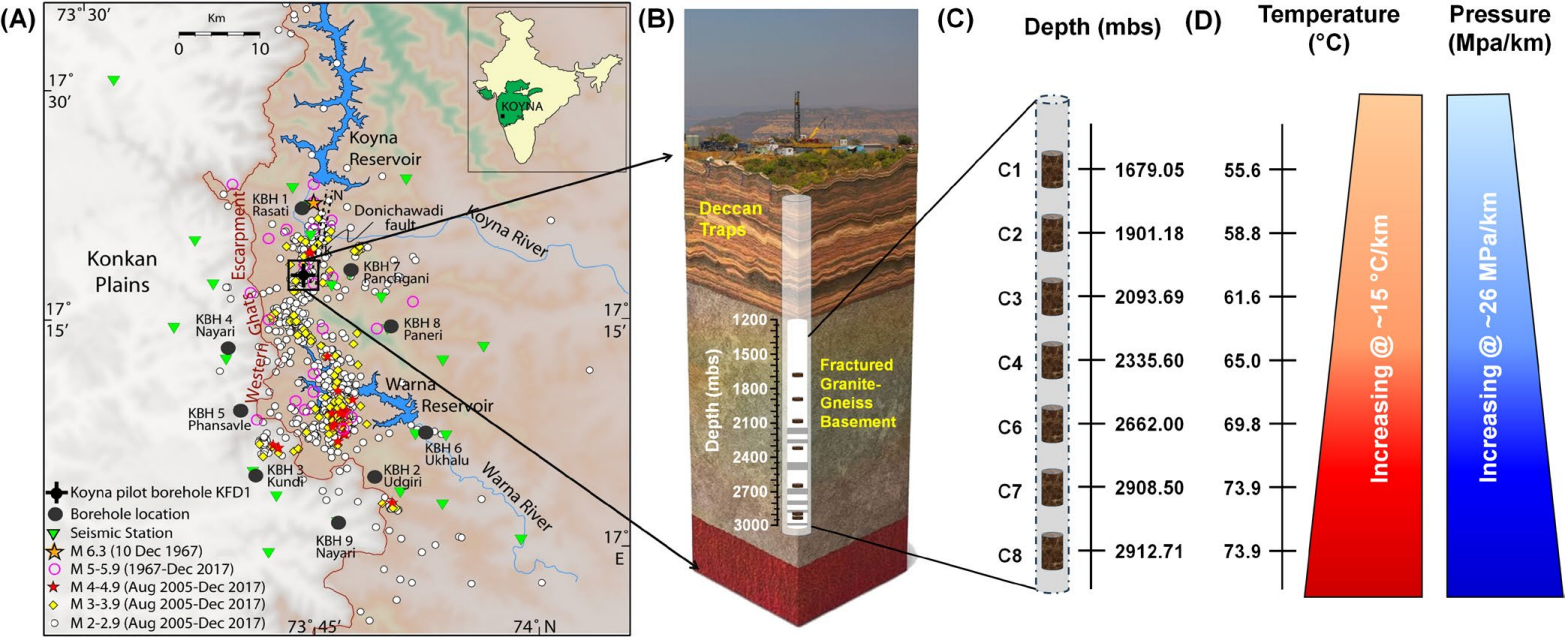
Map of study site displaying (A) location of the 3014 m deep KFD1 borehole drilled at Koyna seismogenic zone of Deccan Traps, India (Inset: Location of Koyna in the Deccan Traps province (shaded green) on the outline map of India) (B) Schematic diagram of the KFD1 borehole passing through upper basaltic layers of the Deccan Traps and placed up to ~3014 m within the Archean granitic basement. Fault damage zones as delineated by Goswami et al. (2019) are highlighted in grey (C) Depths from where the rock cores are obtained. Depth is represented as meters‐below‐surface (mbs) (D) In situ temperature and pressure of the rock samples.

Here, we aim to investigate the ecological processes underpinning microbial communities hosted by the Archean granitic basement beneath the ~65 Ma old Deccan traps, the massive continental flood basalt province of India (Schoene et al. 2015; Courtillot et al. 1986; Duncan and Pyle 1988; Zacharaiah 1998). A 3014 m deep scientific borehole (KFD1) drilled within the Koyna seismic zone (KSZ) of the Deccan Traps at Gothane (17°17′57.27″ N, 73°44′18.07″ E), Maharashtra, India provided a unique opportunity to investigate the deep life present within the crystalline granitic bedrock (Roy 2017). The KFD1 borehole passed through 1247 m thick basalt and continued 1767 m in the underlying granitoid crust. Rock cores were collected during the drilling of the KFD1 at eight depth intervals between 1679 m and 2912 m below the surface, out of which seven were sampled for investigating the deep subsurface microbiology. Detailed location and schematic diagram of the KFD1 borehole is presented in Figure 1. Fault damage zones as delineated through geophysical investigations (Goswami et al. 2019) are highlighted in the same figure (Figure 1B) along with the depth (Figure 1C) and in situ temperature (Figure 1D) from where the rock cores are sampled and used for the deep life study.

With respect to microbial life, Archean granitic basement of the Deccan Traps represents an unusual extreme environment. Progressive increase in temperature and lithostatic pressure [at a rate of ~15°C/km and ~26 MPa/km, respectively] (Roy and Rao 2000; Goswami et al. 2024), low organic carbon (< 0.6 mg TOC/kg) and presence of multiple heavy metals (Ba: 524 mg/kg, Sr.: 323 mg/kg, Cr: 1648 mg/kg, Cd: 15 mg/kg) and radioactive elements (^238^U: 6 mg/kg, ^232^Th: 36 mg/kg) were reported (Dutta et al. 2018; Roy and Rao 2000; Goswami et al. 2024; Sahu et al. 2022; Shukla et al. 2022). In spite of such extremes, this crystalline environment is found to be inhabited by diverse bacterial and archaeal species (Dutta et al. 2018; Dutta et al. 2019). Recently the microbial diversity and function of the crystalline basement were studied using the core samples recovered from the depth range 1600–3000 m in KFD1. A study by Sahu et al. (2022) reported that microbial life is dominated by a few core members via a close interplay between autotrophy and organotrophy. Selective enrichment based reactivation of this rock microbiome led to the identification of chemolithotrophic bacteria (Mandal et al. 2022; Saha et al. 2024). While the presence of diverse and active bacterial populations in the deep, hot, crystalline basement rock is successfully demonstrated, ecological processes controlling the microbial ecology of this extreme realm remain unresolved.

In the present study, the fundamental aspects of microbial ecology of the deep, hot, granitic rock hosted biosphere are investigated. The main objectives of this study are to (i) delineate the species distribution and (ii) determine the environmental variables and ecological processes governing microbial variability and interactions along a depth gradient. These proposed aims are achieved through analysing the 16S rRNA gene sequence data (BioProject ID: PRJNA739059) and geochemical characteristics previously obtained in our laboratory from the rock cores retrieved through KFD1 and used in our earlier investigation (Sahu et al. 2022). Multivariate analyses, phylogenetic distance‐based ecological null models [β‐nearest taxon index (βNTI) and Raup‐Crick (Bray‐Curtis) (RC_BC_)], co‐occurrence networks, and correlation matrices (cohesion matrix) are used to address previously unresolved aspects of this biosphere. Furthermore, the effect of seismic activity on the community assemblage is explored by comparing community cohesion values with helium (Podugu et al. 2019) and acetate concentration, measured in KFD1 borehole fluids. We have hypothesized that increase in extremes and varied geochemical conditions with depth within the deep continental crust have a strong role in shaping subsurface microbial ecosystems by influencing species distribution, community assembly, interaction, complexity, and stability. To our knowledge, this study presents the first report delineating the patterns of species distribution and ecological processes responsible for shaping microbial communities in a seismically active zone of continental Archean basement rock down to a depth of ~3000 m.

## 2 Experimental Procedures

### 2.1 Site Description, Sampling Details, and Sample Processing

Granitic rock core samples used in this study were obtained from crystalline Archean basement present underneath the Deccan Traps through 3014 m deep Koyna pilot borehole (KFD1), drilled at Koyna (17°17′57.27″ N, 73°44′18.07″ E), Maharashtra, India (Figure 1). Drilling and rock core recovery was performed following the standard recommended procedure to minimize the contamination (Kieft et al. 2007; Colwell et al. 2008). In order to assess the possible contamination of rock cores by drilling fluid used during the drilling, sodium fluorescein (500 mg/L) was added to the circulating drilling fluid following the protocol mentioned in Nyyssönen et al. (2014). Details about the site, drilling procedure and sample recovery are described in Sahu et al. (2022) and also mentioned in Supporting Information. Rock cores thus obtained were sub‐sampled aseptically and placed in sterile, N_2_ flushed gas tight bags and stored under anaerobic, low‐temperature (0°C–4°C) conditions in anaerobic jars (Hi‐Media), initially at the drilling site. Samples were shipped to IIT Kharagpur laboratory, maintaining a low temperature (0°C–4°C) in appropriate containers. Along with the rock cores, drilling fluid samples were also collected and transported to the laboratory following the same procedure to monitor drilling fluid contamination in the rock sample.

In the laboratory, rock samples were surface sterilized using 70% ethanol under N_2_ atmosphere. Sub‐coring of rock cores was done only at the central portion of the cores, as recommended to obtain rock powders (Kieft et al. 2007). In order to assess the extent of contamination of the rock cores through the intrusion of drilling fluid and collection of contamination‐free sample, presence of sodium fluorescein was checked in all the rock powder samples collected from each rock core through spectrofluorimeter. Fluorescein rich drilling fluid was used as positive control. For this, 1 g of rock powder or drilling fluid was mixed with 10 mL of deionized water and left overnight on a shaker (150 rpm) under dark conditions. The supernatant was filtered through a 0.22 μm membrane filter, and fluorescence was measured at 540 nm using a spectrofluorimeter (Perkin Elmer). Rock powder samples devoid of any sodium fluorescein were selected for all further microbiological studies. Rock powder and remaining rock cores were stored anaerobically at −70°C in a sterile, DNA‐free container until further geochemical and microbiological analyses. A detailed list of rock samples collected for the study, their depth and in situ temperature (obtained from downhole temperature measurements after drilling) are given (Figure S1).

### 2.2 Geochemical Analysis

Concentrations of anions, major elements and element oxides of the rock samples and ^4^He, CH_4_, H_2_, CO_2_ measured from formation fluids were reported earlier (Sahu et al. 2022; Podugu et al. 2019) and also mentioned in the Supporting Information. CH_3_COO^−^ was measured from borehole water on a Dionex ICS‐1100 ion chromatograph using a six‐point linear calibration and USGS‐206 and USGS‐212 standards for quality control. Uncertainty estimates were based on the standard deviation from three repeat measurements. Concentrations of the measured elements, metal oxides, formation gases, acetate used in this study have been given in Figure S1.

### 2.3 Microbiome Analysis

#### 2.3.1 DNA Extraction, 16S rRNA Gene Amplicon Sequencing and Analysis

16S rRNA gene sequences used in this study were previously obtained from these rock samples and published in Sahu et al. (2022). Essential details are also described here. Total DNA extracted from seven rock cores was used to obtain 16S rRNA gene sequence through amplicon sequencing. Total DNA was also extracted from the reagent control using the same procedure used for 16S rRNA amplicon sequencing and subsequently to check any possible contamination in rock sequence data sets. The detailed description of DNA extraction protocol and 16S rRNA amplicon sequencing has been published in Sahu et al. (2022). Considering the inherent heterogeneity of the crystalline granitic rocks and the presence of possible microhabitats (with different minerals offering potential sites for microbial colonization) within the samples, and in order to capture the microbial diversity to the best possible extent, rock powders were obtained from different portions of the central part of each of the seven rock cores as subsamples used for DNA extraction. In total 49 (7 × 7) subsamples were used to cover the seven samples obtained from depth range 1679–2912 mbs. From each subsample total DNA was extracted multiple times (8 to 10 times) and pooled. V4 region of 16S rRNA gene was amplified from each total DNA pool using barcoded 515F (5′‐GTGCCAGCMGCCGCGGTAA‐3′) 806R (5′‐GGACTACVSGGGTATCTAAT‐3′) primer sets (Bates et al. 2011) and sequenced through Ion S5 (Thermo‐Fisher Scientific) with Ion530 chip. Forward primer was tagged with sample specific 10–12 bp barcode for multiplexing during sequencing run. The PCR was performed in a 25 μL of reaction mixture in triplicates according to the following process: initial denaturation at 95°C for 5 min, 35 cycles of denaturation at 95°C for 45 s, annealing at 50°C for 40 s, elongation at 72°C for 40 s, and final elongation at 72°C for 5 min. The amplicons were held at 4°C. Details of 16S rRNA gene amplicon library preparation and sequencing through Ion S5 (Thermo‐Fisher Scientific) with Ion530 chip were the same as published before Dutta et al. (2018). Total DNA from reagent control was also extracted and sequenced following the same procedure. 16S rRNA gene amplicon sequence from the drilling fluids sampled during the drilling were already obtained in our laboratory and published. The same sequences (BioProject ID: PRJNA482760) were used in this study to check further any possible contamination of the rock derived 16S data sets by drilling fluid derived sequences Bose et al. (2020).

A total of 56 16S rRNA gene amplicon sequence data sets [49 subsurface granitic rock (i.e., seven sequence data sets for each of the seven samples), 06 drilling fluid and 01 reagent control] were used in the analysis. Single‐ended raw reads obtained from Thermo Ion S5 sequencer were analysed using QIIME2 (q2cli v2024.5.0) pipeline and tools therein (Bolyen et al. 2019). Using DADA2 (q2‐dada2 v2024.5.0), the reads were truncated with parameters ‘‐‐p‐trunc‐len 290 ‐‐p‐trim‐left 30 ‐‐p‐trunc‐q 2’, denoised using default parameters, chimera‐filtered with ‘pseudo’ pooling via ‘consensus’ method with ‘‐‐p‐min‐fold‐parent‐over‐abundance 2’ and dereplicated into ASVs (Callahan et al. 2016). Taxonomy was assigned to the ASVs using q2‐feature‐classifier plugin by training it on V4 region (between primers 515F/806R) of SILVA 138 database entries clustered to 99% identity (Bokulich et al. 2018; Robeson et al. 2021; Yilmaz et al. 2014). All the suspected spurious ASVs [singletons (ASVs represented by only one read), ASVs present in reagent control, drilling fluid as well as ASVs affiliated with potential subsurface contaminant microorganisms (Sheik et al. 2018)] were removed from the ASV table. Details of the potential contamination removal procedure have been described in the section below. Data set was normalized to 42,000 reads by random sub‐sampling to equalize sequencing depths in each sample. These normalized sequences were used for rarefaction analyses and calculating various alpha diversity indices viz., Shannon diversity, Ginni‐Simpson, Chao1, Goods‐Coverage, Faith's phylogenetic diversity (PD).

The sequences were submitted to short read archive under BioProject ID: PRJNA739059.

#### 2.3.2 Removal of Potential Contaminants

In order to obtain contamination free sequence data sets, ASVs present in reagent control, ASVs affiliated to potential deep subsurface contaminants [i.e., ASVs affiliated to the members of the taxa *Staphylococcaceae*, *Streptococcaceae*, *Propionibacteriaceae*, *Lactobacillaceae*, and *E. coli‐Shigella* (Sheik et al. 2018)], and dominant ASVs (relative abundance > 0.5%) of drilling fluid [as suggested in (Amils et al. 2023)] were removed from the ASV table.

#### 2.3.3 ASV Occurrence, Multivariate and Variation Portioning Analyses

Occurrence pattern of the ASVs were determined by plotting occurrence of ASVs in samples against their average relative abundance. Spearman correlation between ASV occurrence and average relative abundance was determined. The depth‐wide pattern of microbial communities was determined through the Bray‐Curtis distance‐based Weighted Pair Group Method with Arithmetic Mean (WPGMA) analysis using MVSP 3.2 tool. Detrended Correspondence Analysis (DCA) was performed to determine the appropriate analytical approach between Canonical Correspondence Analysis (CCA) and Redundancy Analysis (RDA) (Chen et al. 2024). As the first axis length of DCA was < 3.0, RDA analysis was chosen as recommended in (Chen et al. 2024). Pair‐wise Spearman correlations among the geochemical variables, individual test using RDA model and Variance Inflation Factor (VIF) analyses were performed to determine the independent environmental variables (׀r׀ < 0.8, VIF < 10) capable of best explaining the community variability. Correlation between the depth‐wide pattern of independent environmental variables and microbial communities was analysed through the RDA and Mantle test. Variance portioning analysis (VPA) was done to evaluate the contribution of environmental variables in explaining community variability. All the above analyses were performed using vegan, Hmisc, dplyr, geosphere, car, ggplot2, ggrepel, and xlsx libraries in R. Similarity percentage (SIMPER) analysis was done using PAST v4.0.3 (Hammer et al. 2001).

#### 2.3.4 Clique Network Analysis

To identify the group of microorganisms responding similarly to the prevailing geochemical conditions a clique network was constructed following the workflow mentioned in Fullerton et al. (2021). In brief, a co‐occurrence network was constructed among the predominant genera (cumulative abundance > 0.5%) of total samples. Only strong positive correlations (*r* > 0.7) were considering for network construction in R using libraries tidyverse, igraph, scales, RColorBrewer. Modularity of the network was determined by all the three algorithms (Random walks, Label propagation and Louvain clustering). Since Random walks algorithm provided best results (highest modularity) it was considered for further identification of the modules. The network was visualizsed through Gephi 0.10.1. Following Fullerton et al. (2021), we have considered each modules as ecological cliques of phylotypes (genera in this case) showing a cohesive distribution across the KSZ subsurface. The relationship between each clique's cumulative abundance and environmental factors was further investigated by Spearman rank correlation.

#### 2.3.5 Ecological Modelling

Phylogenetic bin‐based and distance‐based ecological null models [β‐nearest taxon index (βNTI) and Raup‐Crick (Bray‐Curtis) (RC_BC_)] were used to elucidate impact of different deterministic and stochastic processes on community assembly following the framework developed by Stegen et al. (2013), Stegen et al. (2015), Ning et al. (2020). To equalize the sample depth, samples were rarefied to 350,000 reads by random sampling. Phylogenetic distance between the ASVs was determined by building an unrooted tree using MAFFT v7. Phylogenetic bin‐based null model was analysed using iCAMP R package v1.8.1. Best phylogenetic signal threshold (*d*_s_) and minimum bin size (bin.size.limit *N*_min_) was determined through phylogenetic signal test using ps.bin function. Best phylogenetic signal threshold and minimum bin size (*d*_s_ = 0.05, *N*_min_ = 18) was used for iCAMP analysis using icamp.big function with sig.index = SES.RC, phylo.metric = bMPD options.

During βNTI analysis, phylogenetic turnover between the samples, that is, β‐mean nearest taxon distance (βMNTD) was evaluated for each pair of samples. β‐nearest taxon index (βNTI) was determined as the number of standard deviations of the observed βMNTD from the null βMNTD obtained through community randomization for 999 times. Resulting βNTI values were used to determine the ecological process governing community assembly. If |βNTI| > 2, indicated the role of deterministic processes (environmental selection), and if |βNTI| < 2, indicated the role of stochastic processes in shaping community assembly. Deterministic processes could further be classified into variable selection (βNTI > 2; communities were more different than would be expected by random chance) or homogenizing selection (βNTI < −2; communities were more similar than expected by random chance) (Stegen et al. 2015; Danczak et al. 2018). Similarly, stochastic processes could be dispersal limitation, homogenizing dispersal, and ecological drift. The role of various stochastic processes was further evaluated by an ASV abundance weighted phylogenetic null model, Raup‐Crick (Bray‐Curtis) (RC_BC_) model, following the established framework (Stegen et al. 2015). First, Bray‐Curtis values (BC_obs_) were evaluated for each pair of samples and compared with expected Bray‐Curtis values (BC_null_) obtained through 9999 randomizations to determine the deviations. These deviations were further normalized to vary between −1 and 1 to obtain the Raup‐Crick (Bray‐Curtis) (RC_BC_) matrix. If RC_BC_ > 0.95 implied greater community turnover than expected, indicating the role of dispersal limitation. Similarly, RC_BC_ < −0.95 implied less community turnover than expected, indicating the role of homogenizing dispersal. |RC_BC_| < 0.95 indicated that no single ecological process could explain the community variation. Null models were analysed in R using ape, picante, foreach, abind, readxl, reshape2, iCAMP and doMC libraries.

#### 2.3.6 Co‐Occurrence Network and Cohesion Matrix

Random Matrix Theory‐based co‐occurrence network and correlation matrix (also known as a cohesion matrix) were constructed among all the ASVs with a persistence cutoff of 0.5 (four out of seven subsamples) in a sample to measure community connectivity and complexity. Networks were constructed following the Molecular Ecological Network Analyses Pipeline (http://ieg4.rccc.ou.edu/mena), which is based on Random Matrix Theory (Deng et al. 2012). ASVs with occurrence more than 50% (four out of seven subsamples) were considered for network construction. ASV table was transformed following the cantered log‐ratio method, and missing values were filled with 0.01 if paired valid values were available. For comparison, RMT threshold was set at 0.960 to construct the networks. First, all the three methods: greedy modularity optimization, short random walks and leading eigenvector of the community matrix were used for module separation and modularity calculation. As greedy modularity optimization showed best modularity value, it was used for further module analyses. All the topological features, that is, average degree, network diameter, graph density, modularity, module class, clustering coefficient, and average path length, were calculated using the built‐in functions. Networks were visualized using Gephi 0.10.1. Cohesion analysis was done following published protocols (Danczak et al. 2018; Herren and McMahon 2017).

## 3 Results

### 3.1 Geophysical and Geochemical Properties

Granitoid core samples collected from KSZ presented an overall granite‐gneiss nature. Details of the physical and chemical properties of the samples were described earlier by Sahu et al. (2022), Goswami et al. (2019) and Podugu et al. (2019). In brief, the samples were found to be organic carbon lean (TOC upto 0.6 mg/kg) with presence of various water soluble anions (NO_3_^−^: 54.3 ± 2.8 mg/kg, SO_4_^2−^: 16.7 ± 2.2 mg/kg and NO_2_^−^: 1.2 ± 0.3 mg/kg), elements (Fe: 703 ± 60 mg/kg, Mg: 13.5 ± 0.50 mg/kg, Mn: 20.8 ± 0.7 mg/kg, Ca: 73.6 ± 6.2 mg/kg, Na: 74 ± 11 mg/kg, and K: 100.8 ± 8.3 mg/kg) and metal oxides (Fe_2_O_3_: 41.6% ± 5.4%, SiO_2_: 48.6% ± 2.8%, CaO: 20.0% ± 5.2%, Al_2_O_3_: 11.6% ± 1.7%, and MgO: 2.6% ± 0.4%) in varied range (Sahu et al. 2022). Water flushing of ~100 m intervals following coring runs coupled with online gas analysis (OLGA) during the drilling of KFD1 detected up to 1200 ppmv CO_2_, 186 ppmv CH_4_, 139 ppmv H_2_, and 12.8 ppmv He in crustal gas (Podugu et al. 2019). Zones enriched in gases are mostly below 2100 m with significant He enhancement ranging from 4.6 to 7.6 ppmv above the atmospheric value. In situ geophysical well log data and wellbore images collected during drilling provided compelling evidence that the borehole has cut across localized fault and damage zones, particularly below 2100 m depth (Goswami et al. 2019). The fault damage zones were characterized by anomalous physical and mechanical properties, stress‐induced shear‐wave anisotropy, and rotations in stress orientation, which in turn correlated with anomalous escape of ^4^He gas. The observed geochemical parameters were used in this study to investigate their effect on community variability. Downhole variations in concentrations of anions, elements, metal oxides and formation gases used in this study are also shown in Figure S1.

### 3.2 Sequence Details and Alpha Diversity Parameters

16S rRNA sequence reads retrieved from the granitoid rocks of seven different depths of the deep crystalline basement of the Deccan Traps were subjected to a rigorous, subsample level analysis. A varied number of good quality reads ranging from 46,476 to 451,469 were obtained from 49 libraries [seven libraries per sample; 7 × 7 (details in Tables S1–S7)]. Raw data used in this study are available at NCBI SRA (BioProject ID: PRJNA739059) and previously published by Sahu et al. (2022). The raw data were thoroughly reanalysed [denoising and Amplicon Sequence Variants (ASVs) clustering using DADA2 (Callahan et al. 2016) followed by taxonomic affiliation through Silva 138 database (Yilmaz et al. 2014)] and used in this study. These sequences were clustered into 445–2661 numbers of ASVs (Tables S1–S7). Alpha rarefaction plots of samples' richness approached asymptotic trend (Figures S2, S3), suggesting that our replicated DNA extraction from multiple sub‐samples and sequencing based approach exhaustively sampled the rock microbial diversity. Details of alpha diversity indices were provided in Tables S1–S7. Shannon Diversity Index (D) revealed presence of diverse microbial communities (Figure S4A) with maximum species richness noted in C3 sample (D = 9.48 ± 1.68), and least in C1 (*D* = 7.44 ± 0.71). Gini‐Simpson and Chao1 indices (Figure S4B,C) also indicated the occurrence of highly diverse microbial communities across the depth. PD as estimated via Faith's PD, which identified the C8 community as more diverse and C4 as the least (Figure S4E). Phylogenetic assignment of the ASVs showed presence of 50 taxonomic groups at phylum level. A maximum of 38 phyla were detected in C8 and minimum of 31 phyla in C4 and C6 (Tables S1–S7).

### 3.3 ASV Occurrence Pattern

**FIGURE 2:**
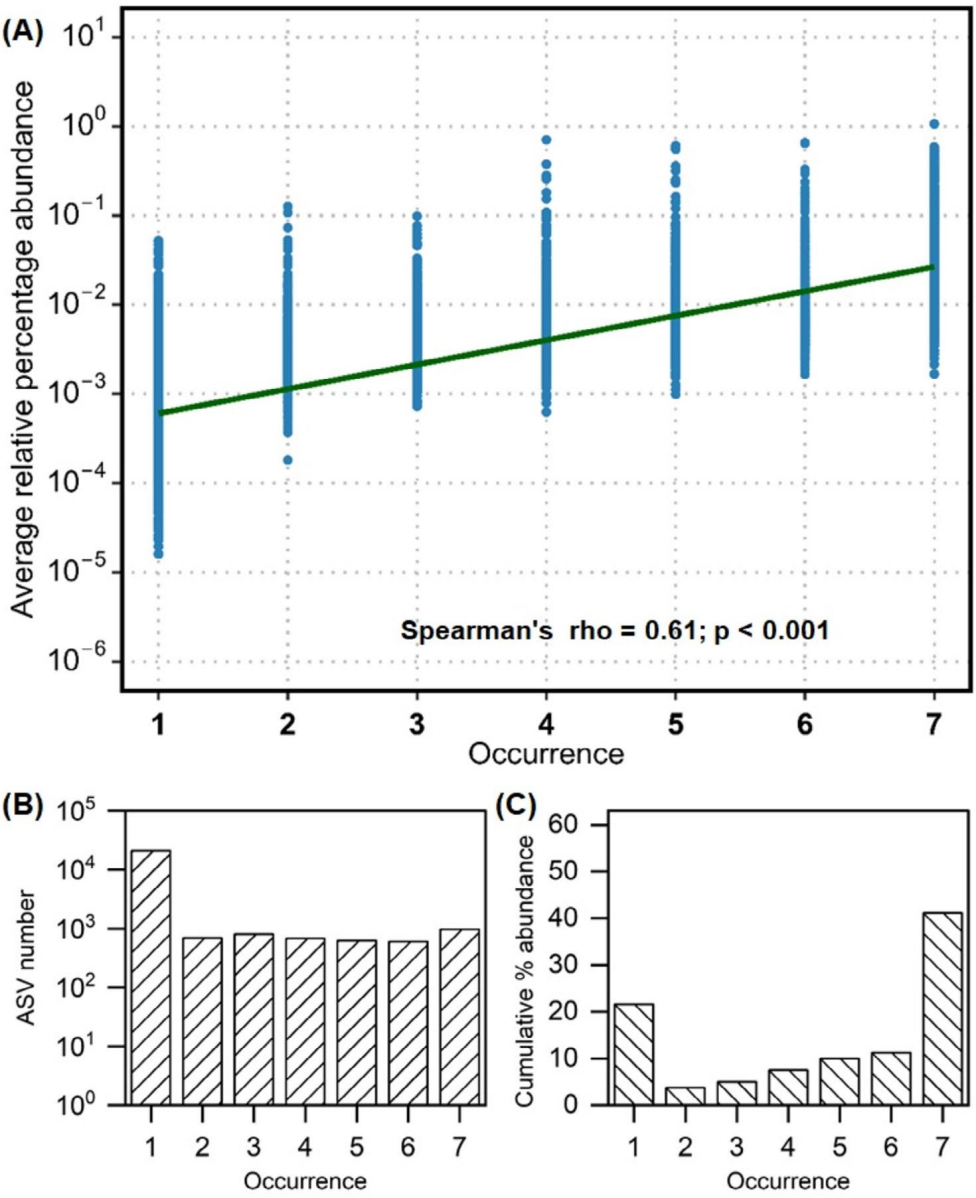
(A) Abundance versus occurrence relationship of all Amplicon Sequence Variants (ASVs) detected across the seven samples of the Archean granitic basement. Spearman rank correlation was calculated between average relative percentage abundance and number of samples occupied by each ASV. (B) Total number of ASVs in each occurrence (C) Cumulative percentage abundance of ASVs in each occurrence.

The distribution of bacterial and archaeal ASVs across the seven samples spanning a depth range of 1679 to 2912 mbs was displayed by an ASV occurrence pattern (Figure 2) to investigate the frequencies of species within the communities across the depth. It is expected that although microbial communities are often constituted by many species, all of these are not equally distributed across the environmental gradients; a few species are assumed to be abundant and more frequently present and many others with lower relative abundance barely appear in the community (Zapién‐Campos et al. 2022). Considering the depth wise gradient of multiple physicochemical parameters and increasing extremes prevalent in the studied deep biosphere (Sahu et al. 2022), the ASV occurrence pattern portrayed the effect of varied environmental conditions on species distribution. A significant positive correlation (rho = 0.61, *p* < 0.001) between the relative abundance and samples occupied by each ASV was observed (Figure 2A). The ASV occurrence plot showed that ASVs with low relative abundances were less frequent and remained confined to any one or two samples only (Figure 2B,C). Approximately 83% ASVs (20,840 out of a total of 25,187 ASVs) were found to be restricted to any one of the seven samples and mostly represented by the rare taxa (ASVs with abundance < 0.01%) with cumulative abundance of only 13%–29%. This was in contrast to the distribution of relatively abundant ASVs, which were found to be more cosmopolitan in nature and well spread across the samples.

### 3.4 Microbial Community Composition and Variability With Depth

**FIGURE 3:**
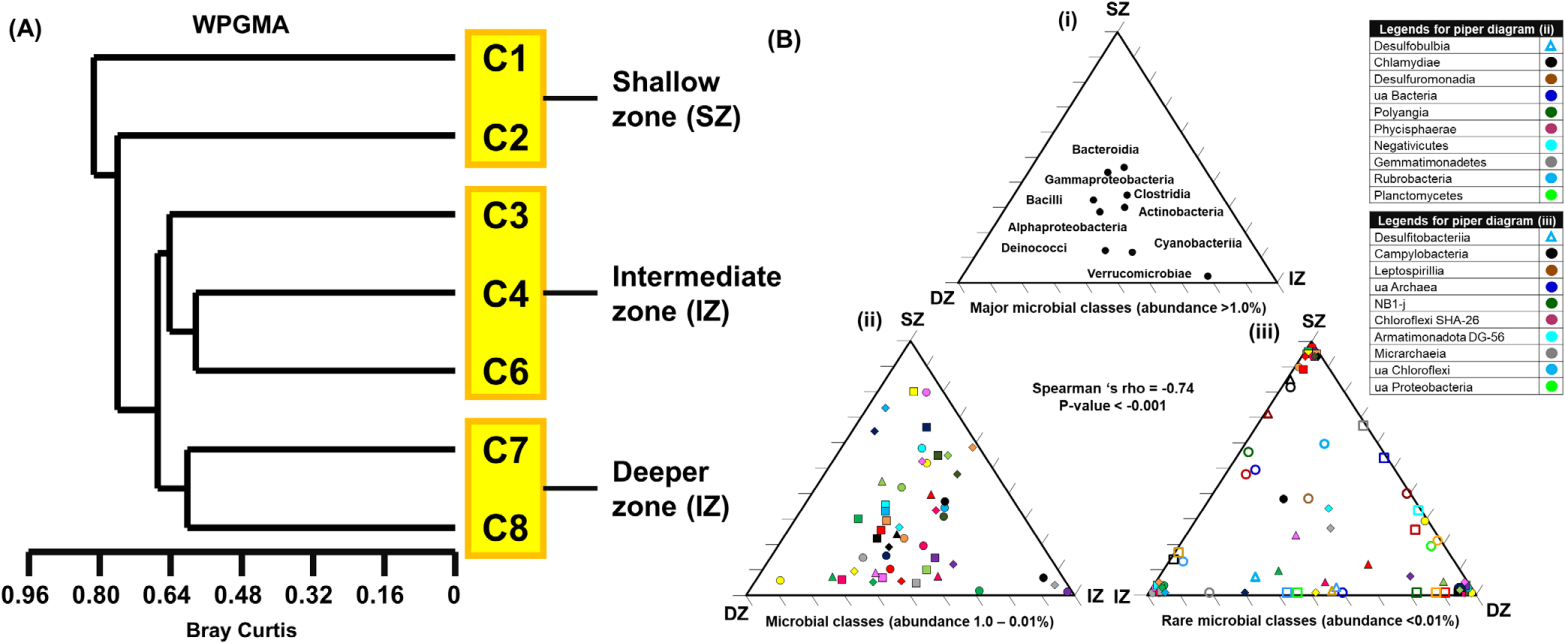
(A) Weighted Pair Group Method with Arithmetic Mean (WPGMA) analysis based clustering of the deep subsurface microbial communities. Three different zones, namely Shallow Zone (SZ), Intermediate Zone (IZ), and Deeper Zone (DZ) are marked. (B) Piper diagram displaying the distribution of microbial classes detected across the three zones of this deep biosphere. Three individual ternary plots presented the relative abundance of major (> 1%) taxa (i), moderately (1.0%–0.01%) abundant (ii), and low (< 0.01%) abundant/rare (iii) taxa. Lists of microbial classes for (ii) and (iii) were provided as Tables S12 and S13, respectively. Spearman rank correlation was calculated between average relative percentage abundance and zone occupied by each microbial class.

Bray‐Curtis distance based WPGMA analysis of all the ASVs showed a clear separation of microbial communities present within seven samples obtained from seven different depths. Presence of similar communities in samples from nearby depths and increase in community dissimilarities with increasing depth were observed (Figure 3A). As evident from the cladogram, microbial communities obtained from seven depths could be partitioned into three distinct clusters (designated hereby as zones): (a) shallow zone (designated as SZ; comprising of C1 and C2 communities), (b) intermediate zone (designated as IZ; C3, C4 and C6 communities) and (c) deeper zone (designated as DZ; C7 and C8 communities) (Figure 3A). PERMANOVA analysis supported this observation showing significant inter‐zone (SZ, IZ, and DZ) variation (*R* ^2^ = 0.40, *p* = 0.051) (Table S8).

Distribution of different bacterial and archaeal lineages across the three zones (SZ, IZ, and DZ) was analysed through Piper diagrams. Three Piper diagrams based on the mean relative abundance of microbial classes [(i), abundance > 1.00%, (ii) abundance 1.00%–0.01%, and (iii) abundance < 0.01%] across the zones were plotted (Figure 3B, Tables S9–S11). A significant (rho = −0.74, *p* < 0.001) pattern of dispersion of microbial taxa was observed. Abundant taxa were mostly equi‐distributed (Figure 3Bi) whereas the taxa with lower abundance were found to be localized in either of the zones (Figure 3Biii). This dispersion pattern of taxa corroborated well with ASV occurrence pattern as presented above (Figure 2). Among the major microbial classes (abundance > 1.0%) *Gammaproteobacteria*, *Actinobacteria*, *Alphaproteobacteria*, *Bacilli*, *Bacteroidia*, and *Clostridia* were distributed more or less evenly across the three zones. *Deinococci*, *Cyanobacteriia*, and *Verrucomicrobiae* were found to be relatively more abundant in IZ and DZ than the SZ (Figure 3Bi). Members of the rare‐microbiome (abundance < 0.01%) represented by diverse classes of bacteria [e.g., *Omnitrophia*, *Sumerlaeia*, *Bacteroidota* SJA‐28, *Altiarchaeia*, *Cloacimonadia*, Bacteria NB1‐j, *Leptospirillia*, *Armatimonadota* DG‐56, *Deferribacteres*, *Micrarchaeia*, *Patescibacteria* ABY1, Chloroflexi SHA‐26, *Entotheonellia*, *Syntrophobacteria*, and so forth, (see Table S11 for more details)] showed a distinct zone‐specific occurrence. SIMPER analysis indicated 21.84% dissimilarities in the distribution of major classes across the three zones whereas more than 75% dissimilarities in distribution of the rare classes (Table S14). Distribution of major genera (mean relative abundance > 0.5%) across the three zones were studied (Figure S5, Table S15). Genera affiliated to *Corynebacterium, Lawsonella, Pseudomonas, Stenotrophomonas, Massilia, Ralstonia, Methylobacterium‐Methylorubrum* and *Anaerococcus* were relatively more abundant in SZ. Opitutaceae IMCC26134, *Shewanella, Pseudonocardia, Kocuria* and *Micrococcus* were more predominant in IZ. The deeper zone (DZ) showed higher abundance of genera *Nocardioides, Comamonas, Enhydrobacter, Sphingomonas, Craurococcus‐Caldovatus* and *Bacillus*.

### 3.5 Effect of Environmental Variables on Microbial Communities

**FIGURE 4:**
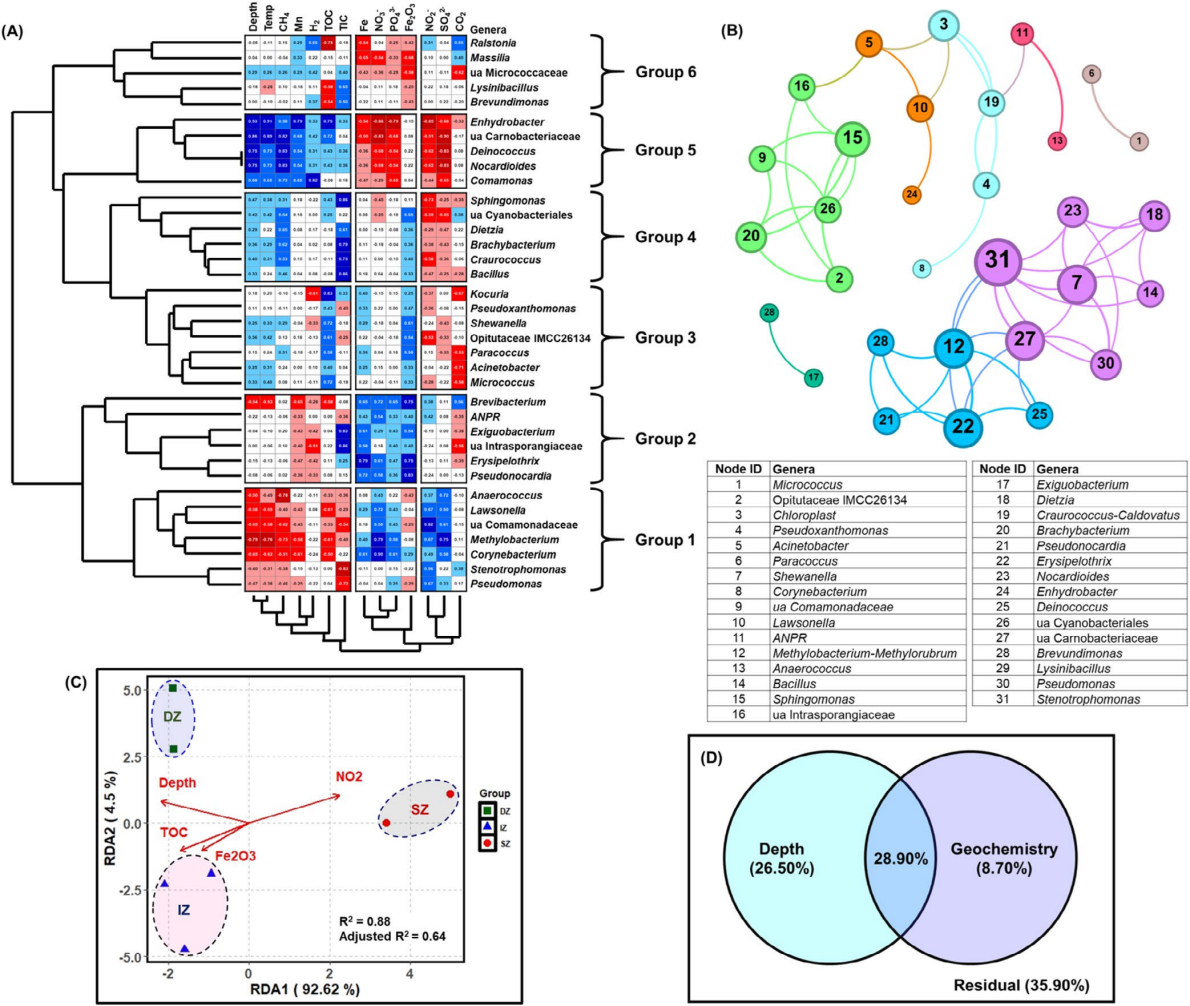
(A) Heatmap and hierarchical clusters displaying the correlation (Spearman) between the dominant genera (mean relative abundance > 0.5%) and the major environmental factors. (B) Co‐occurrence network among the dominant genera. Node colour represented the modular class and size proportional to degree of connections. Following Fullerton et al. (2021), each module of the network is termed as a clique. (C) Redundancy analysis (RDA) depicting the variation in the communities as well as their relationship with the local geochemical parameters. (D) Variation partitioning analysis (VPA) displaying the microbial community variation explained by depth‐wide factors and other geochemical parameters (nonlinear with depth).

Influence of the local environmental variables on the distribution of major bacterial genera (mean relative abundance > 0.5%) was ascertained through pair‐wise Spearman correlation analysis (Figure 4A). Downhole variations in concentrations of elements, metal oxides, formation gases used in this analysis are shown in Figure S1. The analysis identified major environmental drivers controlling the distribution of individual taxa. Based on the down‐core variations in local variables including depth and temperature, clustering of bacterial genera into six different groups was noted. Genera belonging to Group 1 that is, *Anaerococcus*, *Lawsonella*, unassigned (ua) Comamonadaceae, *Methylobacterium*, *Corynebacterium*, *Stenotrophomonas*, *Pseudomonas* were positively correlated (*r* > 0.50) with NO_2_^−^, SO_4_^2−^, NO_3_^−^ and PO_4_^3−^ but negatively correlated (*r* < −0.50) with depth, temperature, CH_4_, Mn, TOC and TIC. Similarly, genera of Group 2 (*Brevibacterium*, *Allorhizobium‐Neorhizobium‐Pararhizobium‐Rhizobium* (*ANPR*), *Exiguobacterium*, unassigned (ua) Intrasporangiaceae, *Erysipelothrix* and *Pseudonocardia*) were positively correlated with Fe, NO_3_^−^, PO_4_^3−^ and Fe_2_O_3_. On the other hand, genera of Group 5 (*Enhydrobacter*, ua Carnobacteriaceae, *Deinococcus*, *Nocardioides*, *Comamonas*) were positively correlated with depth, temperature, CH_4_, Mn, H_2_, TOC and TIC but negatively correlated with Fe, NO_3_^−^, PO_4_^3−^, NO_2_^−^ and SO_4_^2−^. Group 4 genera (*Sphingomonas*, ua Cyanobacteriales, *Dietzia*, *Brachybacterium*, *Craurococcus* and *Bacillus*) showed positive correlation with TIC and CH_4_. Individual genera specific correlation with various parameters were noted for Group 3 and 6 members. *Kocuria* showed a strong positive correlation with TOC (*r* = 0.83) but negative correlations with CO_2_ (*r* = −0.67) and H_2_ (*r* = −0.61). *Shewanella* exhibited positive associations with Fe_2_O_3_ (*r* = 0.61) and TOC (*r* = 0.72). *Opitutaceae* IMCC26134 was correlated positively with TOC (*r* = 0.61) and Fe_2_O_3_ (*r* = 0.54) but negatively with NO_2_^−^ (*r* = −0.53). Similarly, *Paracoccus* displayed positive relationships with TOC (*r* = 0.58) and Fe_2_O_3_ (*r* = 0.50) but an inverse correlation with CO_2_ (*r* = −0.53). *Acinetobacter* was negatively linked to CO_2_ (*r* = −0.71), while *Micrococcus* had a strong positive correlation with TOC (*r* = 0.72) and a negative association with CO_2_ (*r* = −0.58). *Ralstonia* demonstrated a significant positive correlation with TOC (*r* = 0.83) and H_2_ (*r* = 0.66) but correlated negatively with CO_2_ (*r* = −0.67). *Massilia* was negatively associated with Fe (*r* = −0.65) and Fe_2_O_3_ (*r* = −0.68). ub Micrococcaceae showed negative correlations with Fe_2_O_3_ (*r* = −0.58) and CO_2_ (*r* = −0.62). *Lysinibacillus* and *Brevundimonas* exhibited moderate positive correlations with TIC (*r* > 0.5) but negative associations with TOC (*r* < −0.5).

Microbial groups (genera) responding similarly to the prevailing geochemical conditions was further elucidated by co‐occurrence network of major bacterial genera (mean relative abundance > 0.5%) followed by identification of cliques (microbial groups cohesively respond to variation in geochemical parameters across the dataset) following the workflow outlined in Fullerton et al. (2021) (Figure 4B). We identified total eight different cliques of co‐occurring genera. List of genera of each clique has been mentioned in Table S16. Interestingly, most of the cliques contained similar genera grouped together in previous correlation followed by WPGMA analyses (Figure 4A). Response of each cliques to the geochemical factors was further evaluated through the Spearman correlation analysis (Figures S6, S7). Genera of Clique 3 and 6 correlated with most of the geochemical factors either positively or negatively. Clique 1 and 2 loosely correlated with (0.5 < *r < 0.7*) with TOC and Fe_2_O_3_. Clique 3 correlated positively with Fe, NO_2_^−^, NO_3_^−^, SO_4_^2−^ and PO_4_^3−^, and negatively with depth, temperature, TOC, Mn and CH_4_. Clique 4 and 7 correlated strong positively (*r* > 0.9) with TIC. Genera of Clique 5 correlated positively with Fe, Fe_2_O_3_ and NO_3_^−^. Clique 8 correlated positively with NO_2_^−^ (*r =* 0.67) and negatively with TIC (*r =* −0.71).

Influence of various environmental variables on microbial communities was further investigated through RDA. RDA is considered one of the best suited constrained methods to provide direct assessment of how different explanatory variables (environmental variables) contribute to the observed pattern in microbial community variability (Paliy and Shankar 2016). VIF analysis identified depth, TOC, Fe_2_O_3_, and NO_2_^−^ as independent variables for explaining community variability. Details about the prior analyses done for selecting RDA and VIF analyses have been presented as Supporting Information (Figure S8, Table S17–S19).

Significant positive correlation (*r* = 0.51, *p* = 0.02) between the identified independent environmental variables (down‐core variations in depth, Fe_2_O_3_, TOC and NO_2_^−^) and microbial community assemblages was first ascertained through Mantel test. RDA demonstrated that based on prokaryotic community structures and environmental variables, the studied deep biosphere ecosystem can be clustered into three different groups (Figure 4C). The clustering pattern thus obtained resembled the WPGMA based clustering of microbial communities (Figure 3A) confirming the distinct community assemblages across the relatively shallow (SZ), intermediate (IZ) and deeper (DZ) zones of the Koyna deep subsurface. The first two axes of RDA (axis 1 and axis 2) explained 81.41% and 3.95% of variations in the microbial community. NO_2_^−^ was correlated with communities from the shallow zone (viz., C1 and C2). TOC and Fe_2_O_3_ were correlated with intermediate zone communities (C3, C4 and C6) and depth with the communities from the deeper zone (C7 and C8). Variation partitioning analysis (VPA), performed to evaluate microbial community variance explained by the environmental variables further suggested that down‐core changes in depth, TOC, Fe_2_O_3_ and NO_2_^−^ could explain up to 64% of the variations in microbial community composition (Figure 4D, Figure S9). Even though some of the variables significantly affected the community structure, the majority (~36%) of variations in microbial communities were inexplicable by the environmental variables considered in this study. Phylogenetic distance‐based ecological null models were thus applied (next section) to elucidate the impact of different deterministic and stochastic processes on community assembly.

### 3.6 Ecological Processes Controlling Microbial Community Assembly

**FIGURE 5:**
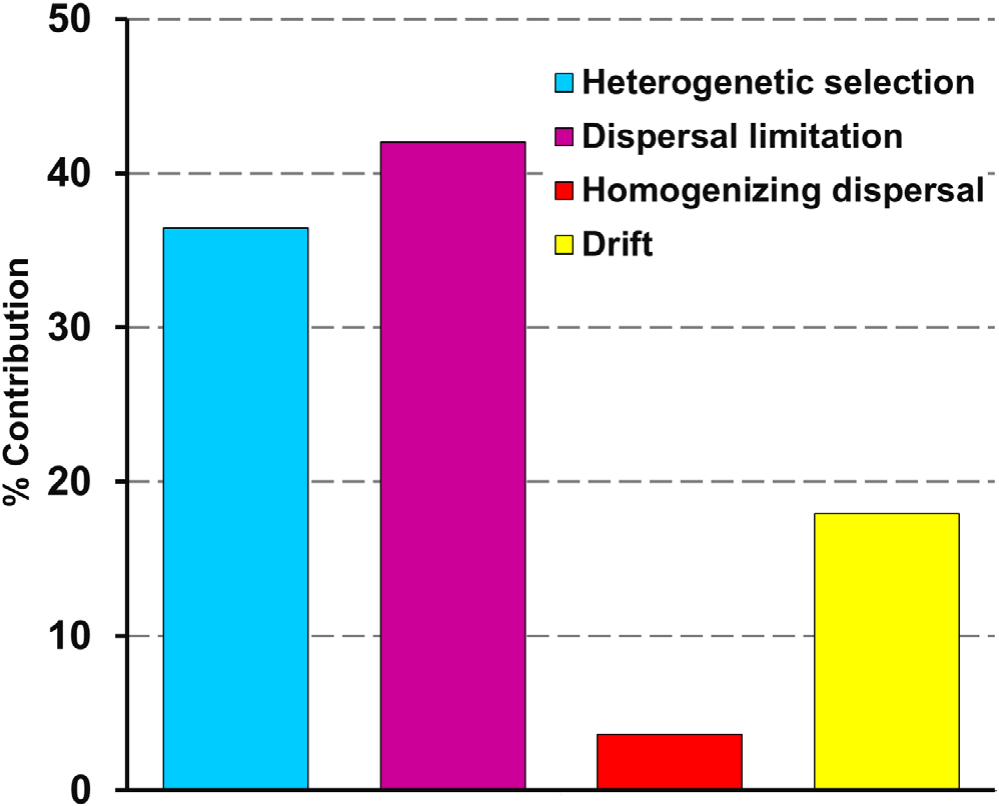
Percentage contribution of ecological processes as resolved through iCAMP analysis.

Relative contribution of different ecological processes (such as deterministic and stochastic processes, as mentioned in the preceding section) in shaping community assembly was ascertained through phylogenetic bin‐based as well distance‐based null models [iCAMP, β‐nearest taxon index (βNTI) and Raup‐Crick (Bray‐Curtis) (RC_BC_)]. Phylogenetic‐bin‐based null model (iCAMP) (Ning et al. 2020) suggested substantial contribution of dispersal limitations (42.02%) and heterogenetic (variable) selection (36.46%) (Figure 5, Table S20). Other stochastic processes like ecological drift and homogenizing dispersal contributed 17.92% and 3.61%, respectively. Phylogenetic distance‐based null models such as β‐nearest taxon index (βNTI) and Raup‐Crick (Bray‐Curtis) (RC_BC_) suggested the contribution of both variable selection (βNTI > 2, RC_BC_ > 0.95) as well as dispersal limitation (|βNTI| < 2, RC_BC_ > 0.95) in driving the community assemblages (Figure S10).

A substantial contribution of dispersal limitation and variable selection suggested an overarching impact of rock petrophysical properties like limited porosity and hydrological connectivity along with niche‐based factors, such as localized abundances of certain nutrients or environmental fluctuations in driving microbial community structure towards a particular composition (Danczak et al. 2018; Zhou and Ning 2017; Huang et al. 2023; Lin et al. 2024). The analyses guided us to infer that KSZ deep biosphere communities exhibited high turnover and less interconnectedness resulting in a less complex community assembly (Danczak et al. 2018).

### 3.7 Community Co‐Occurrence and Interconnectedness Pattern

**FIGURE 6:**
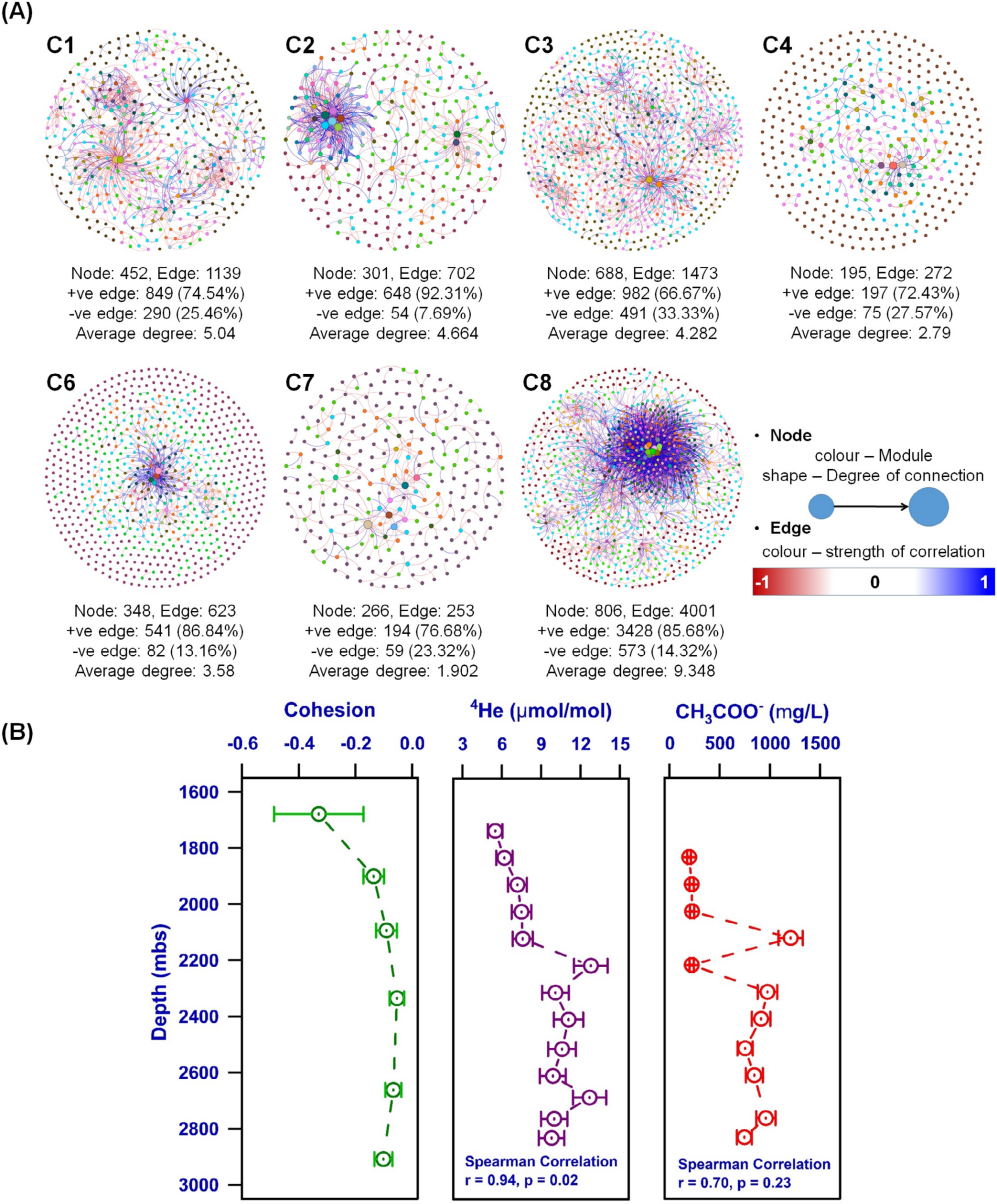
(A) Co‐occurrence networks of the Archean granitic subsurface microbial communities across the depth. Node colour represented the modular class and node size proportional to degree of connections. Edge colour represented the strength of correlation. (B) Depth profiles of community cohesion ^4^He and acetate concentrations ^4^He and acetate were sampled during drilling of KFD1 ^4^He concentration was obtained from Podugu et al. (2019). Weighted pairwise Spearman correlations between depth‐wide pattern of community negative cohesion and ^4^He and acetate concentrations are given as insert.

Co‐occurrence networks and cohesion matrices were used to evaluate the nature of microbial interactions and extent of their interconnectedness across the communities inhabiting different depths. ASVs with persistence cut‐off > 0.5 (ASVs detected in > 50% of the samples) were considered for building Random Matrix Theory based co‐occurrence networks (Figure 6A). Detailed network indices were mentioned in Table S21. The *R* ^2^ of the power law varied between 0.74 and 0.876, indicating the strong and robust, scale‐free network that deviates significantly from a random network (Deng et al. 2012; Liu et al. 2023). Networks consisted of 195–856 nodes and 253–4001 edges with edge to node ratio varying between 0.95 and 4.67. Out of all the connections, 66.67%–92.31% connections were found to be positive in all the networks while 7.69%–33.33% of connections were negative. Higher positive connection suggested a major contribution of mutualistic interactions such as cooperation, cross feeding, co‐colonization and co‐evolution among the microorganisms. Modularity, which measures the strength of division of a network into modules (Newman 2006), showed a variation between 0.365 and 0.890 across the networks (Table S22).

Cohesion matrices showed that cohesion values of the community assemblages vary between negative cohesion (−0.05 to −0.33) and positive cohesion (0.13 to 0.38) (Figure S11). Less negative cohesion values suggested less interconnectedness that is, less complex community assembly (Danczak et al. 2018). A distinct pattern of negative cohesion values across the depth was noted. Highest and lowest negative cohesion values were obtained for C1 (−0.33 ± 0.16) and C4 (−0.05 ± 0.03) communities, respectively.

Cohesion analyses suggested an alteration in community interconnectedness pattern lower negative cohesion values in the intermediate and deeper zones [i.e., 2093–2662 mbs with most pronounced effect in 2335 mbs (C4)] (Figures S11, S12). The altered community behaviour could be explained in the light of the changes in the local geochemical conditions, including fluctuations in the availability of nutrients both from external or endogenous sources, such as influx through fractures. Indigenous microorganisms of this rocky biosphere had possibly responded to these perturbations, thus resulting in the observed shift in community patterns. This observation is in line with the geophysical well log data which strongly indicated the presence of highly fractured zones below 2100 mbs (Goswami et al. 2019). Thus, a possible connection between the shift in microbial community pattern/interaction and the presence of fracture zones emerged. Since the formation of fracture zones in this crystalline environment could partly be attributable to the seismic activity, a connection between microbial communities and seismic activity may also be envisaged.

### 3.8 Possible Links Between Seismicity and the Deep Biosphere at Koyna

In order to delineate the possible influence of subsurface seismic activity on microbial community, negative cohesion values as obtained from communities of different depths were compared with helium (^4^He) and acetate concentrations (indicators for the presence of fracture zones and microbial activity, respectively), sampled from borehole fluids during the drilling of KFD1. The study by Podugu et al. (2019) demonstrated that anomalous levels of ^4^He could be attributed to its preferential inflow through fault/fracture zones into the borehole at different depths. Detailed significance of ^4^He and acetate in this context is described as Supporting Information. ^4^He and acetate concentrations vary in the ranges 34–139 μmol/mol and 195–1208 mg/L, respectively (Figure 6B, Table S23). Significant correlation (rho = 0.94, *p* = 0.02) between the negative cohesion and ^4^He values (Figure 6B) indicate a strong association of fractured zones with community assembly. A weak positive correlation (rho = 0.70, *p* = 0.23) between cohesion and acetate concentration (Figure 6B) further suggests enhanced community activity, especially in terms of the biosynthesis of acetate.

## 4 Discussion

Geological, hydrological, and lithological characteristics of crustal rocks, including heat flow, are considered major drivers of the deep subsurface rock‐hosted microbiome (Templeton and Caro 2023; Magnabosco et al. 2018; Escudero et al. 2018). In this study, we present the details of the role of local environmental variables and ecological forces that govern microbial species diversity, interaction, and assembly within the progressively hot, energy‐limited, crystalline deep rocky biosphere of the KSZ. We show the environmental control of the microbial ecosystem and identify the major drivers that govern this microbial ecosystem. In the following discussion, we evaluate the relationship between the measured environmental variables and some of the important ecological aspects of microbial communities of this deep biosphere.

### 4.1 Influence of Depth‐Wise Environmental Variability on Species Distribution and Community Composition

A distinct pattern in the distribution of microbial species and community composition is observed. ASV occurrence analysis shows significant positive correlations between the abundance of ASVs and their frequency of occurrence across the samples. Relatively large numbers of ASVs with low individual abundances (mainly representing the rare microbiome) remain confined to any one of the samples, indicating their inability to occupy and grow across the depth. On the other hand, the occurrence of the abundant ASVs in multiple samples with varying temperature and other physiochemical conditions indicates their superior metabolic and other abilities. ASV occurrence data are mirrored in the taxonomy‐based Piper diagrams, confirming that abundant taxa of this deep and extreme biosphere are successful across the depth whereas the rare taxa mostly remain localized. The observed distribution pattern of ASVs and bacterial members of diverse phylogenetic lineages highlights the intrinsic characteristics of this deep life. Metabolic state, phenotypic plasticity, and genetic diversity of the inhabitant microorganisms are the major cellular factors affecting the distribution and abundance (an outcome of cell growth) of species across the depth and coupled physicochemical conditions (Nemergut et al. 2013). In response to the physical extremities and energy‐starved state of this deep, rocky biosphere, resident microbial life is expected to proceed at an extremely slow rate (metabolic rate of deep biosphere microorganisms is estimated to be around 10^−5^−10^−3^ fmols C.cell^−1^ d^−1^; 4–5 orders of magnitude lower compared to their surface counterparts) (Jørgensen 2011). A large fraction of the inhabitant microbial community exists in a state of dormancy or anabiosis, devoting most of their metabolic outputs to biomolecular repair and replacement rather than growth (Escudero et al. 2018; Orcutt et al. 2013; D'Hondt et al. 2002). Species which remain dormant or in a suspended metabolism certainly wake up when they are fed, through the supply of necessary nutrients (Jørgensen 2011). It is also highly likely that most species, which exist in a state of anabiosis are actually represented by the rare members, which occasionally get activated and proliferate upon receiving the favourable conditions (may be nutrients in flux due to fracturing, in a seismically active zone, like the KSZ). On the other hand, few metabolically or genomically robust species are able to sustain the chronic stresses and maintain their activities, and are represented by the abundant and frequently detected members across the depth. With respect to utilizing different types of electron donors and acceptors and maintaining cellular homeostasis under varied states of elevated temperature, pressure, ionizing radiation, and so forth, phenotypic plasticity and genetic diversity of individual microbial groups play important roles. They enable microorganisms to survive under extreme conditions and significantly influence the cost of cooperative interaction among the microorganisms for utilizing available nutrients (Hurtado‐Bautista et al. 2021; Chevin and Hoffmann 2017; Kümmerli et al. 2009). Since the supply of different energy resources and environmental stressors changes with varying depth, the ability of the species to adjust their state of metabolism in response remains critical for their distribution across the depth.

Phylogenetic affiliation of most abundant (and cosmopolitan) ASVs suggests predominance of Actinobacteria, Gamma‐, Alpha‐Proteobacteria, *Bacilli*, *Clostridia*, *Cyanobacteria*, *Bacteroidota*, *Verrucomicrobiota* and *Deinococcota*. Multiple geomicrobiological investigations have identified these taxa across different continental subsurface and other extreme environments, highlighting their superior metabolic and genomic potential in thriving within such habitats, obtaining nutrients from local inorganic (mineral) resources and contributing to community resilience (Purkamo et al. 2020; Quraish et al. 2024; Nyyssönen et al. 2014; Magnabosco et al. 2016; Kadnikov et al. 2018; Bell et al. 2020). In consultation with various reports, we infer that a broader swath of plasticity and genetic diversity of the abundant species enables them to thrive even under more extreme and varying electron/carbon‐donor and ‐acceptor regimes and therefore occupy multiple samples. Furthermore, they must be able to spend more energy towards cell growth and having more individuals results in better dispersal events (Liu et al. 2020; Li et al. 2021).

Multivariate analyses indicate that the KSZ deep biosphere within the granitic basement can be grouped into three distinct clusters or zones based on the samples' depth. In general, microbial communities of energy starved deep crystalline rocky habitats are strongly constrained by local geochemical and geophysical (temperature, pressure, porosity, permeability, etc.) parameters (Dutta et al. 2018; Nyyssönen et al. 2014; Dutta et al. 2019; Meyer‐Dombard and Malas 2022; Wu et al. 2023). Paucity of organic carbon necessitates the inhabitant microorganisms to rely strongly on available electron donors and acceptors mainly derived from the minerals, whose nature and availability vary according to the geological and geophysical conditions (Escudero et al. 2018). These varying conditions impose environmental selection for resident microorganisms and drive community assemblage through the development of niches allowing the best utilization of available nutrients (Danczak et al. 2018). With respect to the granitic basement of KSZ, it has been shown earlier that this deep biosphere is deprived of organic carbon, though presence of diverse electron acceptors (e.g., SO_4_^2−^, NO_3_^−^, NO_2_^−^, Fe^3+^, Mn^4+^, etc.) in limited concentrations was observed (Sahu et al. 2022). Apart from temperature and pressure, which show a steady increase across the depth, significant variations in porosity, pH and concentrations of TOC, SO_4_^2−^, NO_3_^−^, PO_4_^3−^, Fe and Mn were also observed (Dutta et al. 2018; Goswami et al. 2019; Sahu et al. 2022). Noticeably, the deep crust as sampled through KFD1 was earlier reported to be partitioned into two geochemically distinct zones: the upper (1679–2335 mbs) comprising of C1, C2, C3 and C4 samples and the deeper zone (2662–2912 mbs) comprising C6, C7 and C8 samples (Sahu et al. 2022). The upper geochemical zone seems to be separated from the deeper one by major fault/fracture zones at multiple depth intervals below 2100 m depth, as delineated on the basis of geophysical and geochemical datasets (Goswami et al. 2019; Podugu et al. 2019). Existence of three distinct microbiological communities in KFD1 basement crust as evidenced in this study, therefore, could be inferred as a direct outcome of the altered geological‐geophysical properties in the intermediate depth (2093–2662 mbs). The presence of open fractures as well as the major cause of the fracture formation, i.e., seismic activity both have strong potential to influence microbial communities. While the seismic activity may promote abiological production of reductants like H_2_ along with various other nutrients (e.g., CO_2_, CH_4_), fracture formation provides habitable space for the cells, and facilitates the flow the fluids and cells (Zhang et al. 2022). All these aspects have a strong impact on the inhabitant microbial communities in terms of species selection, their metabolism, eventually their distribution, abundance and interaction by providing conduit to fluid (Wiersberg and Erzinger 2008; Wiersberg and Erzinger 2011) and nutrient flow, as well as by supplying two important reductants for microbial metabolism, that is, H_2_ and CH_4_. Elevated levels of H_2_ and CH_4_ in borehole fluid has been recently reported from the KFD1 (Podugu et al. 2019). Based on the overall geological‐geophysical characteristics, it is inferred that while the upper and deeper zones have their own drivers for inhabitant microbial communities, the intermediate zone allows a distinct community composition, based on its intrinsic parameters. As a result of this, the overall deep biosphere microbial ecosystem is partitioned into three distinct zones.

Environmental selection of taxa across the depth and their niche partitioning are clearly observed from the hierarchical clustering (Figure 4A). Distribution of these major taxa across the different zones highlights the profound role of environmental selection allowing these populations to occupy various niches based on their physiological and metabolic abilities. Extreme stress tolerant, H_2_ utilizing organisms [e.g., Actinobacteria, *Comamonas* and other members of Burkholderiales (*Ralstonia*), Cyanobacteria and Verrucomicrobia (IMCC26134), etc.] equipped with both autotrophic and heterotrophic CO_2_ fixation mechanisms are selected in the deeper and intermediate zones. *Comamonas* and *Pseudomonas*, along with other members of Burkholderiales are recently identified as keystone members of Fennoscandian deep groundwater and KSZ deep biosphere core community (Soares et al. 2023; Purkamo et al. 2016). A recent report on the presence of the Wood‐Ljungdahl pathway in metagenome assembled genomes of *Comamonas* confirms its ability to fix carbon with minimal energy expenditure (Zhang et al. 2024). Together with this autotrophic ability, presence of dissimilatory nitrate reduction, and assimilatory sulfate reduction pathway contribute to its overall fitness and survival (Zhang et al. 2024). Predominance of Cyanobacteria and IMCC26134 (Verrucomicrobia) in the intermediate zone is validated by their ability to use organic carbon and alternate sources of energy like H_2_, CH_4_, and so forth, (Escudero and Amils 2023; Puente‐Sánchez et al. 2018; Cerbin et al. 2022; Nixon et al. 2019; Dunfield et al. 2007) likely sourced from the fault zone, with higher H_2_ and CH_4_ levels (Goswami et al. 2019; Podugu et al. 2019). *Deinococcus*, a well‐known extremely radiation tolerant taxon, capable of anaerobic growth using Fe(III), Cr(VI), U(VI), and Tc(VII) as electron acceptor have been recovered from 1500 to 2000‐m deep hot springs in Iceland, where temperatures range from 76°C to 91.4°C (Marteinsson et al. 2001). Actinobacteria, present in much higher abundance in the deeper zone, are members of the KSZ endemic community (Sahu et al. 2022). Members of this taxon are known poly‐extremotrophs (Bull and Goodfellow 2019). Their ability to survive extreme temperature, prolonged desiccation, radiation exposure and most notably their metabolic versatility towards utilizing diverse carbon and energy substrates and facilitating interspecies interactions through metabolite transfer make them the most relevant denizens of the extreme rocky biosphere (Bull and Goodfellow 2019). Dominance of actinobacterial members in KSZ deep biosphere as a part of the endemic community is considered to be beneficial in isolated deep biosphere environments where concentrations of different carbon substrates fluctuate over time (Sahu et al. 2022). In comparison to the deeper zone, the shallow zone has shown the abundance of diverse NO_3_^−^, NO_2_^−^, SO_4_^2−^ metabolizing taxa such as *Pseudomonas, Corynebacterium, Stenotrophomonas, Pseudonocardia, Erysipelothrix, Lawsonella, Allorhizobium‐Neorhizobium‐Pararhizobium‐Rhizobium* (ANPR), ua Intrasporangiaceae, *Methylobacterium‐Methylorubrum, Exiguobacterium, Anaerococcus* indicates their colonization in such depths (Purkamo et al. 2016; Nishimura et al. 2007; Kundu et al. 2014; Guo et al. 2024; Lollar et al. 2019; Idris et al. 2017; Purkamo et al. 2015; Tüccar et al. 2019). *Pseudomonas* was previously identified as a keystone member of Fennoscandian deep groundwater and KSZ rocky biosphere (Soares et al. 2023; Sahu et al. 2022). Positive correlation between *Pseudomonas* and NO_3_^−^ corroborated with its key role in local N‐cycling through NO_3_^−^ reduction (Soares et al. 2023; Purkamo et al. 2016; Purkamo et al. 2015). *Exiguobacterium* is an anaerobic, polyextremophilic organisms, highly adaptive to extreme saline (EC ≈ 50 mS/cm) and alkaline (pH 8.5 to pH 10) conditions, and a broad temperature range (−12°C to 55°C) (Ma et al. 2023). These microorganisms were reported to co‐exist with sulphate reducing bacteria and assist in sulphate reduction (Tüccar et al. 2019). Significant positive correlation between *Corynebacterium*, ANPR, *Methylobacterium‐Methylorubrum* and *Stenotrophomonas* corroborates with their potential for NO_3_^−^ reduction as reported previously from diverse extreme environment including hyper‐arid soil of Atacama Desert, subsurface hot brines in northwest Poland, heavy‐oil reservoirs of Russia (Nishimura et al. 2007; Guo et al. 2024; Idris et al. 2017; Kalwasińska et al. 2020; Semenova et al. 2020).

It is interesting to note that although the measured environmental variables are able to elucidate the role of such factors in impacting community composition, a significant portion (~36%) of the community variations remains unexplained (VPA based observation). This highlights the significant contribution of unmeasured geochemical factors such as local lithology that is, presence or absence of fractures, recurrent seismic activity and biological interactions including cooperation, competition, predation, immigration, migration, viral lysis on community assemblages, which mostly remained unassessed (Huang et al. 2023; Lin et al. 2024; Yan et al. 2020).

Phylogenetic bin‐based ecological null models suggest that dispersal limitation, variable selection, and ecological drift are the primary ecological forces governing microbial community assembly in KSZ deep biosphere. Prevailing extremities, lack of pore space [neutron porosity 0.1%–1% (except in fracture zones) (Goswami et al. 2019)] and hydrological connectivity, as well as varying availability of electron‐donor, acceptors and carbon sources in the overall oligotrophic environment of this crystalline biosphere explain the importance of dispersal limitation and variable selection in governing community assembly. Lack of hydrological connectivity, mostly due to the existence of impermeable thick granitic crust, hinders the movement and mixing of microorganisms, leading towards high compositional turnover (Zhang et al. 2022; Danczak et al. 2018). Similarly, varying physicochemical conditions enable niche‐specific selection of species with desirable metabolic traits and facilitate interspecies interactions necessary for survival and flourishing over time (Templeton and Caro 2023; Lin et al. 2024). All of these conditions could also result in high phylogenetic turnover of microbial assemblages across the depth (Templeton and Caro 2023; Danczak et al. 2018; Huang et al. 2023; Lin et al. 2024). Among the various local physicochemical parameters, the availability of nutrients across the depth can be one of the most relevant factors regulating community assembly. In energy‐starved deep crystalline environments, nutrient availability has been known to exert a considerable impact on community assembly. As per the ‘hunger games hypothesis’, in an oligotrophic environment, microbes mitigate the constraints of nutrient limitation by promoting cooperative interaction (relationship) like metabolite exchange for mutual benefit, efficient nutrient utilization, and productivity (Lin et al. 2024; Dai et al. 2022). Cooperative relationships allow the inhabitant community to minimize diversity loss and enhance their stability, as well as shape their assembly by acting as a selection force (i.e, niche differentiation) (Lin et al. 2024; Calcagno et al. 2017).

The cooperative mode of interactions among the KSZ deep biosphere organisms is further evident through co‐occurrence analysis. Co‐occurrence networks across the depth showing predominance of positive connections suggest presence of multimodal mutualistic association (through cooperation, cross feeding, co‐colonization and co‐evolution) among the microorganisms, rather than competition (Huang et al. 2023; Lin et al. 2024). All these positive interactions essentially facilitate more interspecies connections, thus enabling efficient resource and information transfer and stronger resilience of the community to environmental constraints (Lin et al. 2024). Moreover, the observed paucity of negative cohesion values suggests low level of community complexity, further indicating a greater degree of internal dynamics (Danczak et al. 2018). All these corroborate well with the dominant role of dispersal limitation and variable selection highlighted above. A positive correlation of cohesion pattern with ^4^He and acetate concentration measured from the formation fluid together with significant role of ecological drift (random processes) and presence of fracture zones at that depth, as obtained through geophysical logs (Goswami et al. 2019) highlight some additional aspects. Considering the inherent seismic nature of the site, it can be envisaged that recurrent seismic activity might have contributed to occurrence of those fractures or changed their geometry and allowed movement of crustal fluids, gases, and other nutrients, thereby inducing fluctuations in local environment and nutrient dilution. In response to such perturbations, enhanced positive interactions occurred in the microbial community allowing the inhabiting microorganisms to derive nutrients from the surroundings efficiently and resist environmental change/heterogeneity. Thus, such perturbations may have influenced community assembly and interaction to share resources, transfer information, maintain homeostasis and withstand environmental change.

## 5 Conclusion

This study unravels the foundational principles of microbial ecosystem hosted by energy‐ and nutrient‐limited deep terrestrial biosphere within the Archean granitoid basement beneath the ~65 Ma Deccan Traps, India. The study provides new insights into the environmental drivers, assembly processes, interaction patterns and complexity of the microbial communities across the depths of this seismically active crystalline biosphere. Metataxonomic analysis reveals the presence of diverse bacterial communities with universal occurrence of dominant ASVs while rare ASVs remain sample specific, highlighting the limited ability of a large fraction of the total ASVs to migrate along and colonize the depths. Multivariate and ecological null model analyses depict a pronounced effect of limited dispersion and varied environmental conditions in governing community assembly. The analyses guided the inference that deep Koyna Seismic Zone biosphere communities were impacted by both stochastic and deterministic processes. Co‐occurrence network and cohesion analysis show that the communities are inclined to reduce negative interaction and tend to be involved in cooperative interactions for efficient utilization of available nutrients and withstanding the environmental extremities. Significant correlation between community cohesion pattern and ^4^He and acetate concentrations detected in crustal fluids extracted during drilling suggests a potential influence of deep fault/fracture zones on the microbial community assemblages in specific regions of this deep biosphere. A strong association of such deep fault/fracture zones with the seismic activity in the region needs to be further investigated. Overall, our study reveals that the diverse microbial community residing in the Archean granitic basement is shaped by limited dispersion, variable environmental conditions and strong mutualistic interactions among them.

## Author Contributions

**Rajendra Prasad Sahu:** conceptualization, data curation, formal analysis, investigation, methodology, validation, visualization, writing – original draft, writing – review and editing, software. **Sufia Khannam Kazy:** conceptualization, writing – review and editing. **Debarshi Mukherjee:** methodology, writing – review and editing. **Sukanta Roy:** validation, investigation, writing – review and editing. **Thomas Wiersberg:** investigation, methodology, writing – review and editing. **Pinaki Sar:** conceptualization, methodology, validation, resources, writing – original draft, writing – review and editing, supervision, project administration, funding acquisition.

## Funding

This work was supported by the Ministry of Earth Sciences (MoES), Government of India, MoES/P.O.(Seismo)/1(288)/2016 dated 16 March 2017.

## Conflicts of Interest

The authors declare no conflicts of interest.

## Supporting information


**Figure S1:** Down core change in temperature, major rock geochemical parameters and depth profile of concentration of various gases measured from formation fluids during drilling of KFD1. Temperature and rock geochemical parameters data was adopted from Sahu et al. (2022). Bore hole gas data was adopted from Podugu et al. (2019).
**Figure S2:** Alpha rarefaction plot showing the number of observed ASVs as a function of sequencing depth.
**Figure S3:** Alpha rarefaction plot depicting Shannon index as a function of sequencing depth.
**Figure S4:** Alpha diversity [(A) Shannon, (B) Gini‐Simpson, (C) Chao1, (D) Goods coverage and (E) Faith's Phylogenetic Diversity] indices of each community.
**Figure S5:** Distribution of major genera (abundance > 0.5%) across the three zones. Detailed list of major genera and their abundance in respective zones is presented as Table S15. *Allorhizobium‐Neorhizobium‐Pararhizobium‐Rhizobium* is abbreviated as ANPR, uncultured bacterium is abbreviated as ub.
**Figure S6:** Line‐plot displaying the abundance of different cliques and geochemical factors in each sample.
**Figure S7:** Heatmap displaying pairwise spearman correlation between cliques and major geochemical factors.
**Figure S8:** Variance Inflation Factors (VIF) of values of independent geochemical variable considering depth as response variable and TOC, Fe_2_O_3_ and NO_2_^−^ as predictor variables.
**Figure S9:** Variation portioning analysis displaying the microbial community variation explained by depth‐wide factors and other geochemical parameters (nonlinear with depth).
**Figure S10:** Heatmap displaying β‐nearest taxon index [βNTI (blue to green—lower triangle)] and Raup‐Crick (Bray‐Curtis) [RC_BC_ (pink to red—upper triangle)] between the microbial communities present within the Archean granitic basement. Deterministic processes (left side of heatmap) include variable selection (blue; βNTI > 2) and homogenizing selection (green; βNTI < −2). When |βNTI| < 2, the phylogenetic relatedness between two communities did not differ more significantly than expected by chance, and stochastic processes dominate (right side of heatmap). Stochastic processes include homogenizing dispersal (red; |βNTI| < 2 and RC_BC_ < −0.95), dispersal limitation and drift (purple; |βNTI| < 2 and RC_BC_ > 0.95), and undominated (|RC_BC_| < 0.95) processes. (B) Percentage contribution of ecological processes was represented as a barplot.
**Figure S11:** Box‐Whisker plot displaying (A) positive cohesion and (B) negative cohesion of the community obtained from each depth. Cohesion values were determined by considering all the ASVs with persistence cut‐off > 0.5 in each sample.
**Figure S12:** Depth‐wide pattern of –ve cohesion and network topologies (% of +ve and –ve edges).
**Table S1:** Sequence read details and alpha diversity parameters of C1 subsamples.
**Table S2:** Sequence read details and alpha diversity parameters of C2 subsamples.
**Table S3:** Sequence read details and alpha diversity parameters of C3 subsamples.
**Table S4:** Sequence read details and alpha diversity parameters of C4 subsamples.
**Table S5:** Sequence read details and alpha diversity parameters of C6 subsamples.
**Table S6:** Sequence read details and alpha diversity parameters of C7 subsamples.
**Table S7:** Sequence read details and alpha diversity parameters of C8 subsamples.
**Table S8:** Permutational analysis of variance (PERMANOVA) explaining the significant variability between the microbial communities.
**Table S9:** List of microbial classes with mean relative abundance > 1% and their relative abundance in all three zones (SZ: Shallow Zone, IZ: Intermediate Zone, DZ: Deeper Zone).
**Table S10:** List of microbial classes of abundance 0.01%–1% and their relative abundance in all three zones (SZ: Shallow Zone, IZ: Intermediate Zone, DZ: Deeper Zone).
**Table S11:** List of rare microbial classes (mean relative abundance < 0.01%) and their relative abundance in all three zones (SZ: Shallow Zone, IZ: Intermediate Zone, DZ: Deeper Zone).
**Table S12:** Symbols assigned against the microbial classes of mean relative abundance 0.01%–1% in Piper diagram Figure 3Bii.
**Table S13:** Symbols assigned against the rare microbial classes (mean relative abundance < 0.01) in Piper diagram Figure 3Biii.
**Table S14:** Similarity Percentage (SIMPER) analysis explaining dissimilarities between the distribution of microbial classes across three zones.
**Table S15:** List of major microbial genera (mean relative abundance > 0.5%) and their abundance in three zones.
**Table S17:** Detrended Correspondence Analysis (DCA) of microbial communities.
**Table S18:** Pairwise spearman correlation among the geochemical parameters.
**Table S19:** Explainability of each independent geochemical variable.
**Table S20:** Relative importance of each ecological process in different samples. HeS: Heterogeneous selection, HoS: Homogeneous selection, DL: Dispersal limitation, HD: Homogenizing dispersal, DR: Drift and others.
**Table S21:** Detailed topology of each network.
**Table S22:** Modularity of each network.
**Table S23:** Concentration of acetate measured from formation fluid during drilling of KFD1 and its depth‐wide pattern.

## Data Availability

Raw 16S rRNA gene sequence reads are submitted in the NCBI (BioProject number: PRJNA739059). These were obtained through our earlier published study (Sahu et al. 2022, Environ Microbiol 24:2837 ‐ 2853). In this study, raw 16S rRNA gene sequence reads are throughly reanalysed to address the previously unresolved questions aspects of microbial ecology within this biosphere.
