# Supplementary material for: Ecological Processes Underpinning Microbial Variability in Archean Granitoids Beneath the Deccan Traps: Evidence From Deep Drilling in Koyna, India
Source: Environ Microbiol Rep. 2026 May 3;18(3):e70351. doi: 10.1111/1758-2229.70351 (PMC13136517; doi:10.1111/1758-2229.70351)
Supplement: Supplementary file 1 — Figure S1: Down core change in temperature, major rock geochemical parameters and depth profile of concentration of various gases measured from formation fluids during drilling of KFD1. Temperature and rock geochemical parameters data was adopted from Sahu et al. (2022). Bore hole gas data was adopted from Podugu et al. (2019). Figure S2: Alpha rarefaction plot showing the number of observed ASVs as a function of sequencing depth. Figure S3: Alpha rarefaction plot depicting Shannon index as a function of sequencing depth. Figure S4: Alpha diversity [(A) Shannon, (B) Gini‐Simpson, (C) Chao1, (D) Goods coverage and (E) Faith's Phylogenetic Diversity] indices of each community. Figure S5: Distribution of major genera (abundance > 0.5%) across the three zones. Detailed list of major genera and their abundance in respective zones is presented as Table S15. Allorhizobium‐Neorhizobium‐Pararhizobium‐Rhizobium is abbreviated as ANPR, uncultured bacterium is abbreviated as ub. Figure S6: Line‐plot displaying the abundance of different cliques and geochemical factors in each sample. Figure S7: Heatmap displaying pairwise spearman correlation between cliques and major geochemical factors. Figure S8: Variance Inflation Factors (VIF) of values of independent geochemical variable considering depth as response variable and TOC, Fe2O3 and NO2 − as predictor variables. Figure S9: Variation portioning analysis displaying the microbial community variation explained by depth‐wide factors and other geochemical parameters (nonlinear with depth). Figure S10: Heatmap displaying β‐nearest taxon index [βNTI (blue to green—lower triangle)] and Raup‐Crick (Bray‐Curtis) [RCBC (pink to red—upper triangle)] between the microbial communities present within the Archean granitic basement. Deterministic processes (left side of heatmap) include variable selection (blue; βNTI > 2) and homogenizing selection (green; βNTI < −2). When |βNTI| < 2, the phylogenetic relatedness between two communities did not [file EMI4-18-e70351-s001.docx]

**Supplementary Information for**

**Ecological processes underpinning microbial variability in Archaean granitoids beneath the Deccan Traps: Evidence from Deep Drilling in Koyna, India**

Rajendra Prasad Sahu^1^, Sufia Khannam Kazy^2^, Debarshi Mukherjee^1^, Sukanta Roy^3^, Thomas Wiersberg^4^, and Pinaki Sar^1^*

^1^Environmental Microbiology and Genomics Laboratory, Department of Bioscience and Biotechnology, Indian Institute of Technology Kharagpur, Kharagpur, 721302, WB, India

^2^Department of Biotechnology, National Institute of Technology Durgapur, Durgapur, 713209, WB, India

^3^Ministry of Earth Sciences, Borehole Geophysics Research Laboratory, Karad, 415114, India

^4^ GFZ Helmholtz Centre for Geosciences, Telegrafenberg, 14473 Potsdam, Germany

*Corresponding author:

Pinaki Sar, Department of Bioscience and Biotechnology, Indian Institute of Technology Kharagpur, Kharagpur, 721302, WB, India; Email: [psar@bt.iitkgp.ac.in](mailto:psar@bt.iitkgp.ac.in), Phone: +913222 283754, Fax: +913222 255303

**Experimental Procedures**

**Site description, sampling details, and sample processing**

Granitic rock core samples used in this study were obtained from crystalline Archean basement present underneath the Deccan Traps through 3000 m deep Koyna pilot borehole (KFD1), drilled at Koyna (17°17’57.27” N, 73°44’19.07” E), Maharashtra, India (Figure 1). Details about the site and its selection, geological significance, drilling procedure, and sample recovery can be found elsewhere ^1–3^. In brief, drilling of KFD1 was performed following a combination of air hammer and mud rotary drilling. Air hammer technique was used except at a few depths where fluid loss or water ingress zones were encountered and at depths where coring was performed, mud rotary drilling was performed. The borehole KFD1 passed through 1247 m thick Deccan trap and continued 1768 m in the underlying granitic basement rock. Nine-metre-long rock cores were collected at eight depth intervals between 1679 m and 2912 m below the surface. Out of the total eight cores obtained during this drilling, seven were sampled for investigating the deep subsurface microbiology. Details of the seven rock cores used in the study was presented in Figure 1. Drilling and rock core recovery was performed following the standard recommended procedure to minimize the contamination ^4,5^. In order to assess the possible contamination of rock cores by drilling fluid used during the drilling, sodium fluorescein (500 mg/L) was added to the circulating drilling fluid following the protocol mentioned in Nyyssönen *et al.,* (2014) ^6^. Intact rock cores thus obtained were sampled aseptically [with surface-sterilized (using 70% ethanol) granite rock cutter and chisel] on site immediately after recovery of the rock core from the core barrel. All rock core samples were placed in sterile, N_2_ flushed gas tight bags and stored under anaerobic, low-temperature (0 - 4 °C) conditions in anaerobic jars (Hi-Media) initially at the drilling site. Samples were transported to our laboratory (at IIT Kharagpur) maintaining a low temperature (0 - 4 °C) in appropriate shipping containers.

**Geochemical analysis**

Details of rock samples processing and analytical methods used for geochemical characterisation were previously reported ^7^. In brief, major elements of the rock cores were determined by acid digesting one gram of rock sample in a microwave digester (Milestone SK 12) followed by measuring in ICP-MS (iCAP-Q, Thermo Scientific). To investigate major anions (Cl^-^, NO_2_^-^, SO_4_^2-^, NO_3_^-^ and PO_4_^3-^) rock powders were ultrasonicated in deionized water (1:10 w/v) and quantified using Dionex ICS-2100 (Thermo Scientific). Quantification of total inorganic carbon and total organic carbon was performed using OI analytical TOC analyser. Elemental oxides were measured using PANalytical Epsilon3 XRF instrument. All the analyses were performed in triplicate.

**Results**

**Effect of environmental variables on microbial communities**

Influence of various local geochemical/environmental factors on the rock hosted microbial communities was investigated further through a number of statistical tests. To determine the suitability between RDA and CCA (two common tests often used for such analyses) in providing the most appropriate information on our test samples, Detrended Correspondence Analysis (DCA) was performed on microbial community data. Length of the first axis of DCA [DCA1, 0.9871 (Table S17)], suggested the suitability of RDA for explaining community variability ^8^. In order to determine the independent environmental variables capable of best explaining the community variability, pair-wise Spearman correlations among the geochemical variables, individual test using RDA model and Variance Inflation Factor (VIF) analyses were performed. Based on correlations values between the environmental variables (Table S18), depth, temperature, and concentrations of Mn, Fe, NO_3_^-^, SO_4_^2-^, PO_4_^3-^, CH_4_ and H_2_ were identified as collinear variables (׀rho׀ > 0.80). Depth, TIC, TOC, Fe_2_O_3_, NO_2_^-^ and CO_2_ were detected as independent variables (׀rho׀ < 0.80). Explainability of each of these independent variables were detected through individual test using RDA model (Table S19). Among these, depth, TOC, Fe_2_O_3_ and NO_2_^-^ were identified to be most appropriate for explaining community variability. VIF values (< 10) for these variables (Figure S8), further suggested their suitability as environmental variables in RDA analysis ^8,9^.

**Potential influence of seismic activity**

Cohesion measures the interconnectedness of a microbial community (determined as negative cohesion values; higher negative cohesion value = greater community interaction). In this study we have considered anomalous He escaped through interconnected fractured horizons at depth as a proxy for seismic activity and abundance of acetate as a measure of microbial metabolism. Both He and acetate levels were quantified from the borehole fluids immediately after the completion of the drilling. Helium gas is a noble gas that consists of two isotopes: ^3^He (which is mainly of primordial origin) and radiogenic ^4^He from radioactive decay of ^235^U, ^238^U and ^232^Th ^3^. The isotopic ratio ^3^He/^4^He is distinct for different reservoirs (sublithospheric, asthenospheric and deep mantle, continental crust, and air), but rarely exceeds a value of 10^-5^. In the case of Koyna it was >10^-7^ ^3^. ^4^He production takes place in the matrix of uranium and thorium bearing minerals, from where it accumulates in pore space and fractures. Thus enhanced He concentrations in the subsurface can be used as an indicator for the presence of fracture zones that might be formed due to seismic activity. On the other hand, acetate is an important metabolite of microbial metabolism. It can be produced biologically through fixation of inorganic carbon *i.e.* CO_2_ (via Wood-Ljungdahl pathway), or via other metabolic reactions such as incomplete oxidation/degradation of organic compounds ^10,11^. It can be further assimilated as a carbon source into biomass or consumed by heterotrophs ^12^.

**Figure and Table legends**

**Figure S1** Down core change in temperature, major rock geochemical parameters and depth profile of concentration of various gases measured from formation fluids during drilling of KFD1. Temperature and rock geochemical parameters data was adopted from Sahu *et al.,* (2022)^7^. Bore hole gas data was adopted from Podugu *et al.,* (2019)^3^

**Figure S2** Alpha rarefaction plot showing the number of observed ASVs as a function of sequencing depth.

**Figure S3** Alpha rarefaction plot depicting Shannon index as a function of sequencing depth.

**Figure S4** Alpha diversity [**(A)** Shannon, **(B)** Gini-Simpson, **(C)** Chao1, **(D)** Goods coverage and **(E)** Faith’s Phylogenetic Diversity] indices of each community

**Figure S5** Distribution of major genera (abundance > 0.5%) across the three zones. Detailed list of major genera and their abundance in respective zones is presented as Table S15. *Allorhizobium-Neorhizobium-Pararhizobium-Rhizobium* is abbreviated as ANPR, uncultured bacterium is abbreviated as ub.

**Figure S6** Line-plot displaying the abundance of different cliques and geochemical factors in each sample.

**Figure S7** Heatmap displaying pairwise spearman correlation between cliques and major geochemical factors.

**Figure S8** Variance Inflation Factors (VIF) of values of independent geochemical variable considering depth as response variable and TOC, Fe_2_O_3_ and NO_2_^-^ as predictor variables**.**

**Figure S9** Variation portioning analysis displaying the microbial community variation explained by depth-wide factors and other geochemical parameters (nonlinear with depth).

**Figure S10** Heatmap displaying β-nearest taxon index [βNTI (blue to green – lower triangle)] and Raup-Crick (Bray-Curtis) [RC_BC_ (pink to red - upper triangle)] between the microbial communities present within the Archean granitic basement. Deterministic processes (left side of heatmap) include variable selection (blue; βNTI > 2) and homogenizing selection (green; βNTI < -2). When |βNTI| < 2, the phylogenetic relatedness between two communities did not differ significantly than expected by chance, and stochastic processes dominate (right side of heatmap). Stochastic processes include homogenizing dispersal (red; |βNTI| < 2 and RC_BC_ < -0.95), dispersal limitation and drift (purple; |βNTI| < 2 and RC_BC_ > 0.95), and undominated (|RC_BC_| < 0.95) processes. (**B**) Percentage contribution of ecological processes was mentioned as barplot.

**Figure S11** Box-Whisker plot displaying **(A)** positive cohesion and **(B)** negative cohesion of the community obtained from each depth. Cohesion values were determined by considering all the ASVs with persistence cut-off > 0.5 in each sample.

**Figure S12** Depth-wide pattern of –ve cohesion and network topologies (% of +ve and –ve edges).

**Table legends**

**Table S1** Sequence read details and alpha diversity parameters of C1 subsamples

**Table S2** Sequence read details and alpha diversity parameters of C2 subsamples

**Table S3** Sequence read details and alpha diversity parameters of C3 subsamples

**Table S4** Sequence read details and alpha diversity parameters of C4 subsamples

**Table S5** Sequence read details and alpha diversity parameters of C6 subsamples

**Table S6** Sequence read details and alpha diversity parameters of C7 subsamples

**Table S7** Sequence read details and alpha diversity parameters of C8 subsamples

**Table S8** Permutational analysis of variance (PERMANOVA) explaining the significant variability between the microbial communities.

**Table S9** List of microbial classes with mean relative abundance >1% and their relative abundance in all three zones (SZ: Shallow Zone, IZ: Intermediate Zone, DZ: Deeper Zone)

**Table S10** List of microbial classes of abundance 0.01 - 1% and their relative abundance in all three zones (SZ: Shallow Zone, IZ: Intermediate Zone, DZ: Deeper Zone)

**Table S11** List of rare microbial classes (mean relative abundance < 0.01%) and their relative abundance in all three zones (SZ: Shallow Zone, IZ: Intermediate Zone, DZ: Deeper Zone)

**Table S12** Symbols assigned against the microbial classes of mean relative abundance 0.01 - 1% in piper diagram Figure 3B(ii)

**Table S13** Symbols assigned against the rare microbial classes (mean relative abundance < 0.01) in piper diagram Figure 3B(iii)

**Table S14** Similarity Percentage (SIMPER) analysis explaining dissimilarities between the distribution of microbial classes across three zones.

**Table S15** List of major microbial genera (mean relative abundance > 0.5%) and their abundance in three zones.

**Table 16** List of genera in each clique

**Table S17** Detrended Correspondence Analysis (DCA) of microbial communities

**Table S18** Pairwise spearman correlation among the geochemical parameters

**Table S19** Explainability of each independent geochemical variable

**Table S20** Relative importance of each ecological process in different samples. HeS: Heterogeneous selection, HoS: Homogeneous selection, DL: Dispersal limitation, HD: Homogenizing dispersal, DR: Drift and others.

**Table S21** Detailed topology of each network

**Table S22** Modularity of each network

**Table S23** Concentration of acetate measured from formation fluid during drilling of KFD1 and its depth-wide pattern.

**
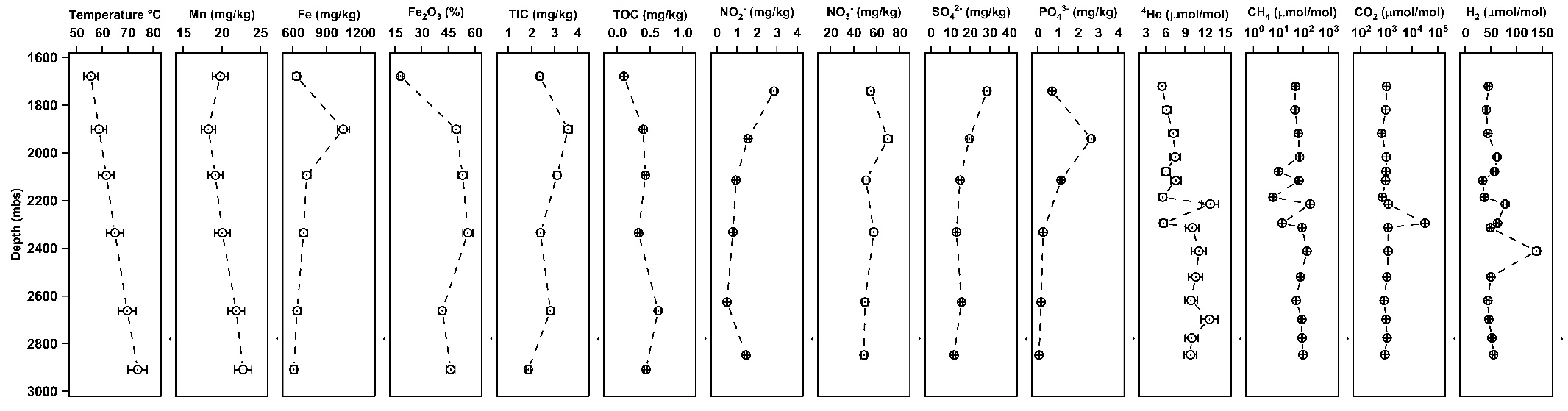
**

**Figure S1** Down core change in temperature, major rock geochemical parameters and depth profile of concentration of various gases measured from formation fluids during drilling of KFD1. Temperature and rock geochemical parameters data was adapted from Sahu *et al.,* (2022)^7^. Bore hole gas data was adapted from Podugu *et al.,* (2019)^3^

**
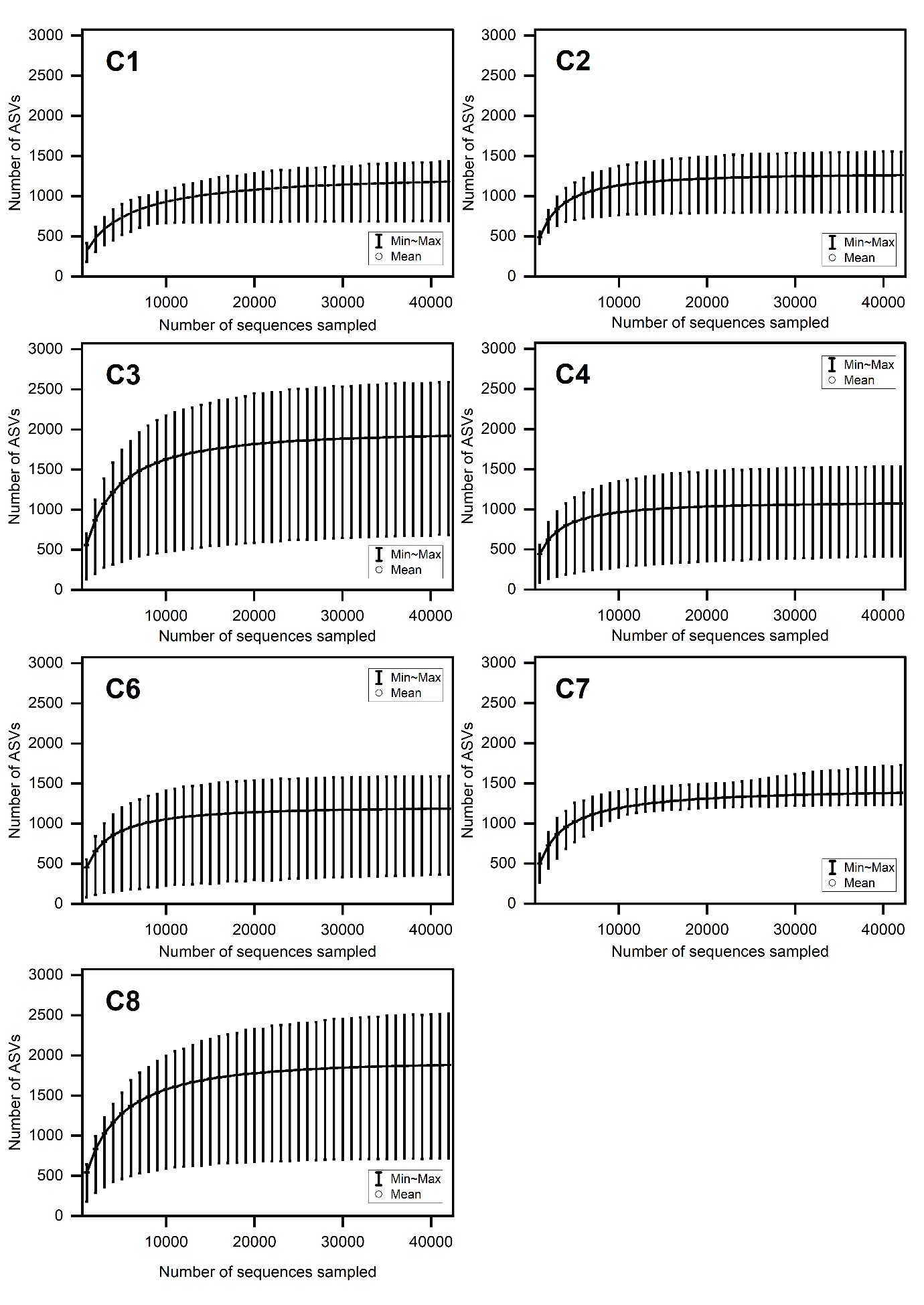
**

**Figure S2** Alpha rarefaction plot showing the number of observed ASVs as a function of sequencing depth.

**
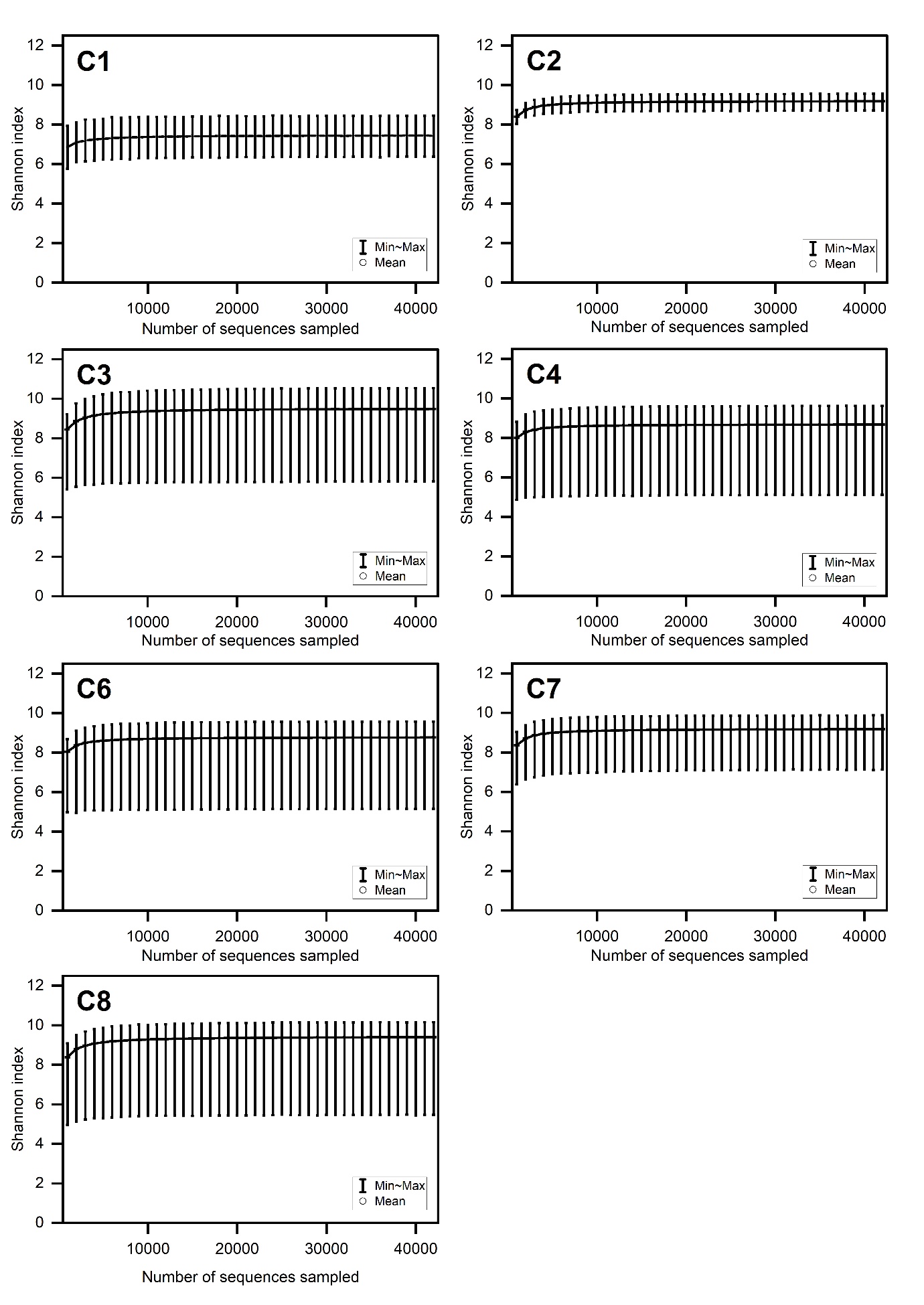
**

**Figure S3** Alpha rarefaction plot depicting Shannon index as a function of sequencing depth.

**
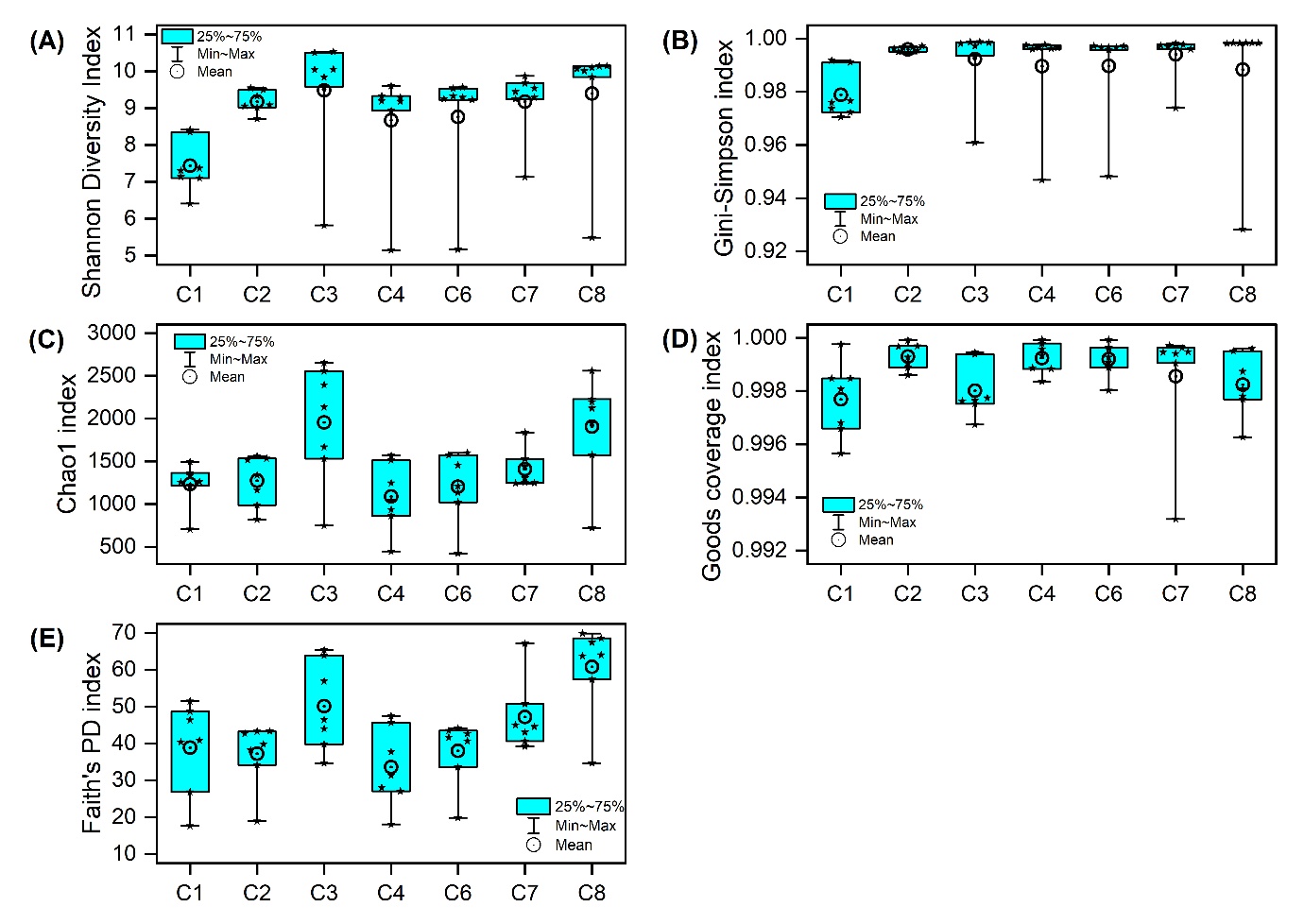
 Figure S4** Alpha diversity [**(A)** Shannon, **(B)** Gini-Simpson, **(C)** Chao1, **(D)** Goods coverage and **(E)** Faith’s Phylogenetic Diversity] indices of each community

**
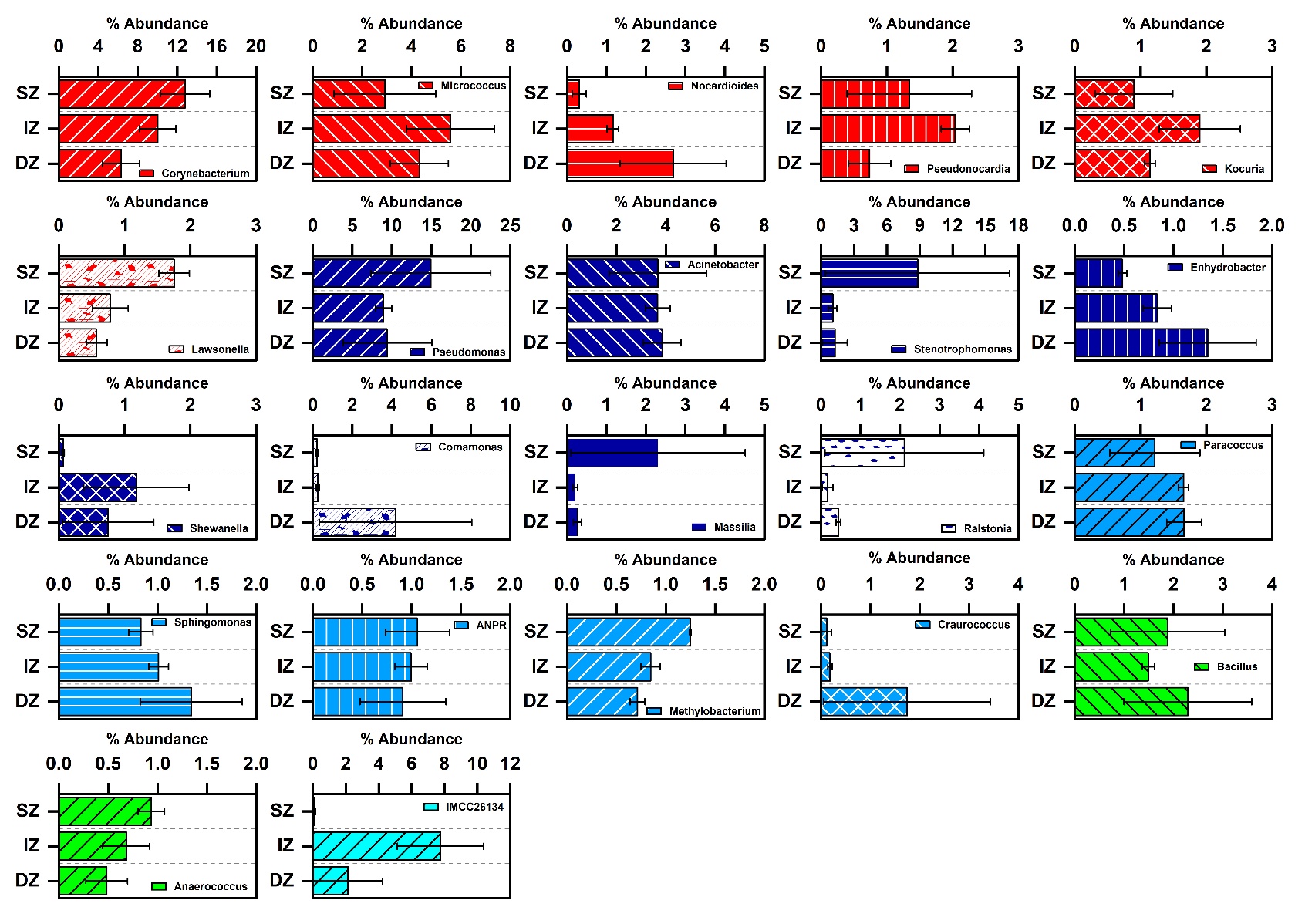
**

**Figure S5** Distribution of major genera (abundance > 0.5%) across the three zones. Detailed list of major genera and their abundance in respective zones is presented as Table S15. *Allorhizobium-Neorhizobium-Pararhizobium-Rhizobium* is abbreviated as *ANPR,* uncultured bacterium is abbreviated as ub.


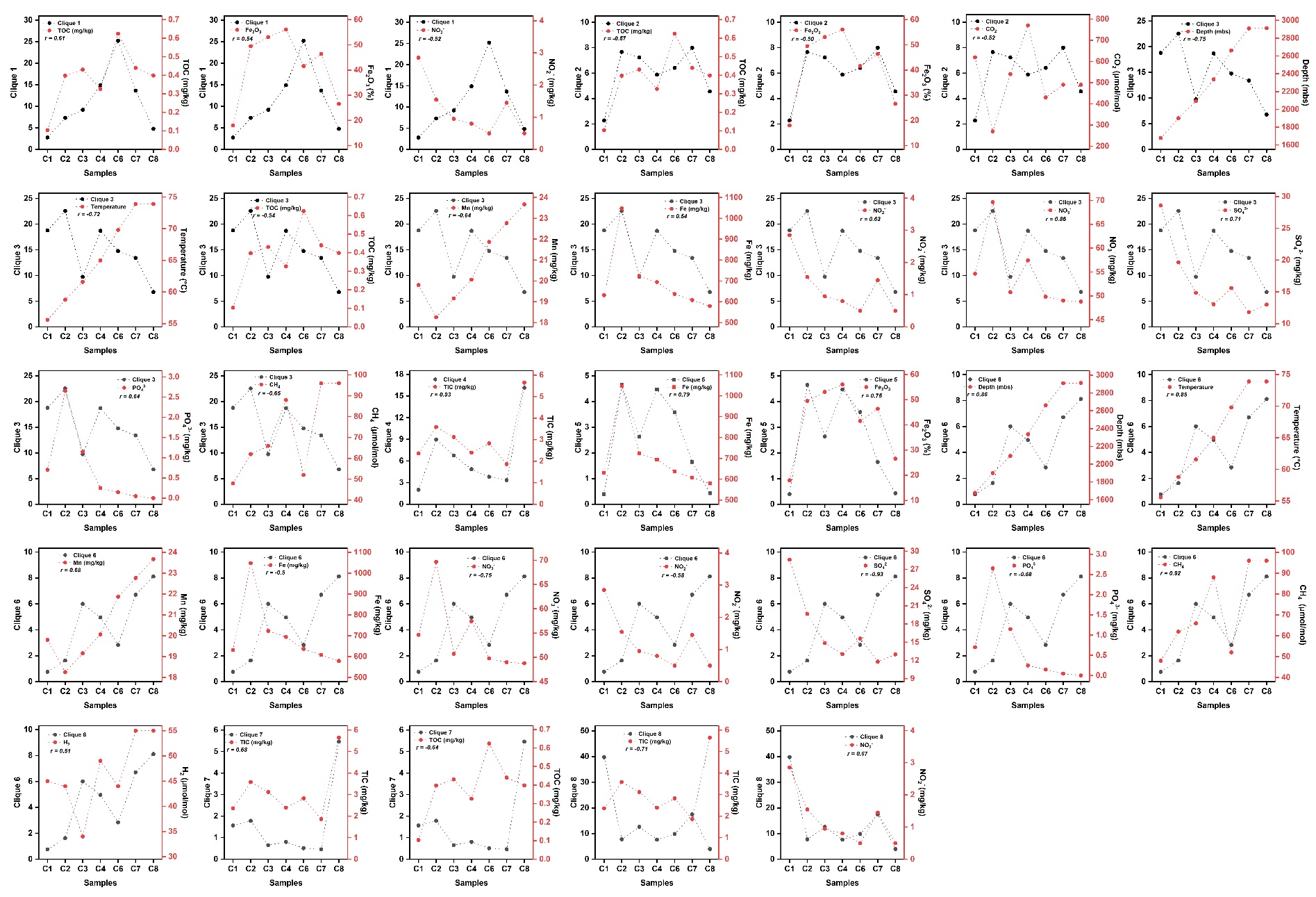


**Figure S6** Line-plot displaying the abundance of different cliques and geochemical factors in each sample


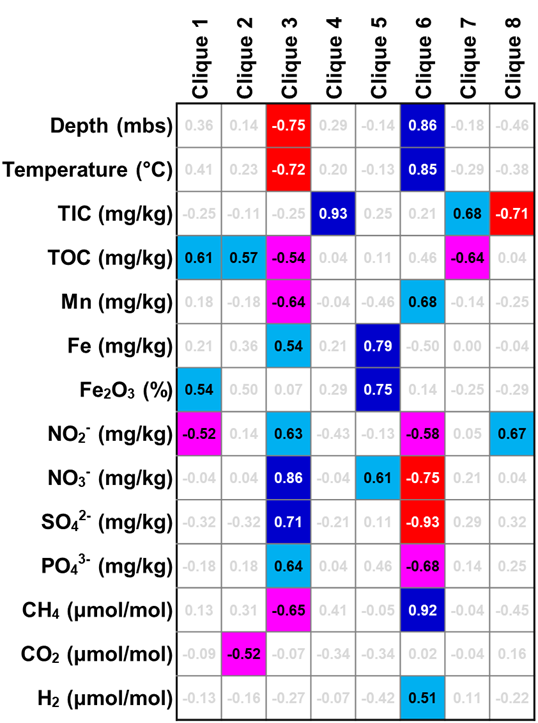


**Figure S7** Heatmap displaying correlation between cliques and major geochemical factors

**
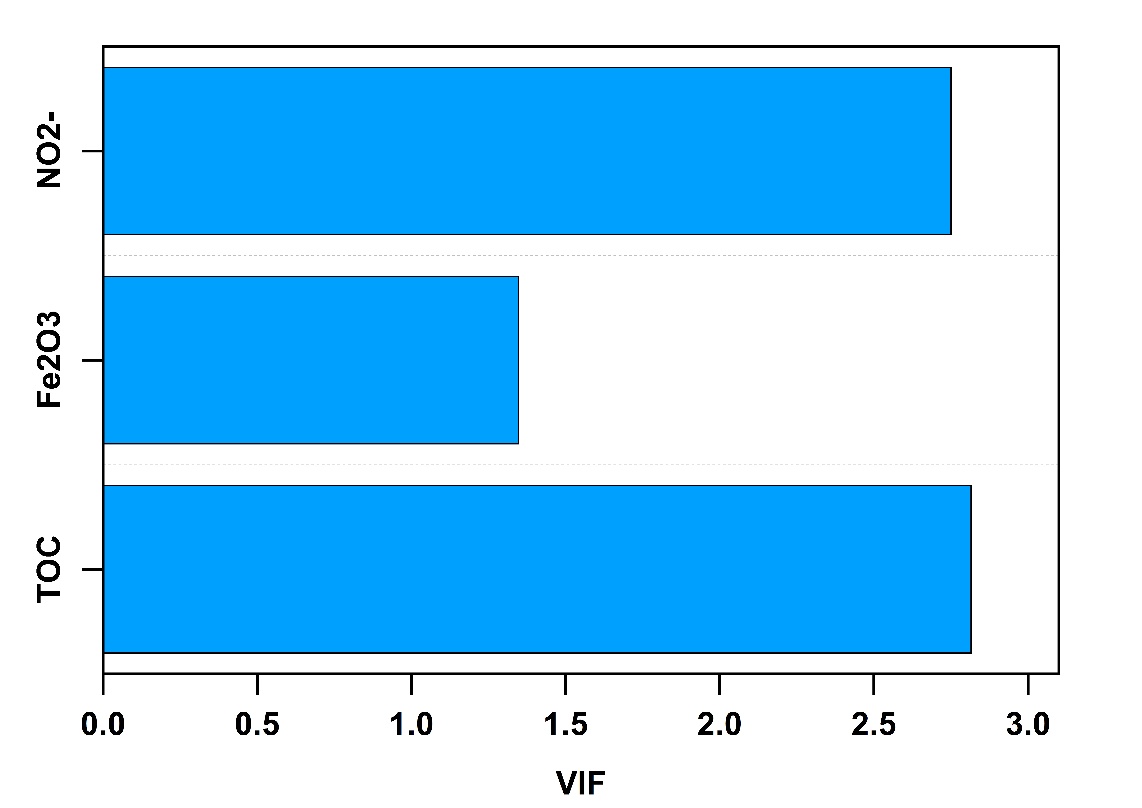
**

**Figure S8** Variance Inflation Factors (VIF) of values of independent geochemical variable considering depth as response variable and TOC, Fe_2_O_3_ and NO_2_^-^ as predictor variables.

**
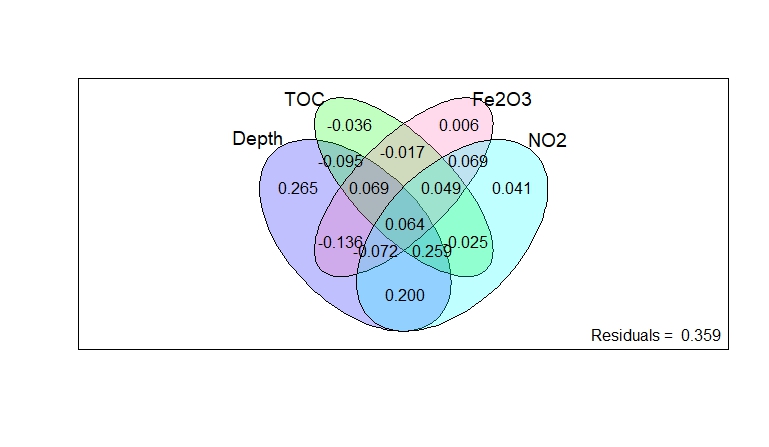
Figure S9** Variation portioning analysis displaying the microbial community variation explained by depth-wide factors and other geochemical parameters (nonlinear with depth).


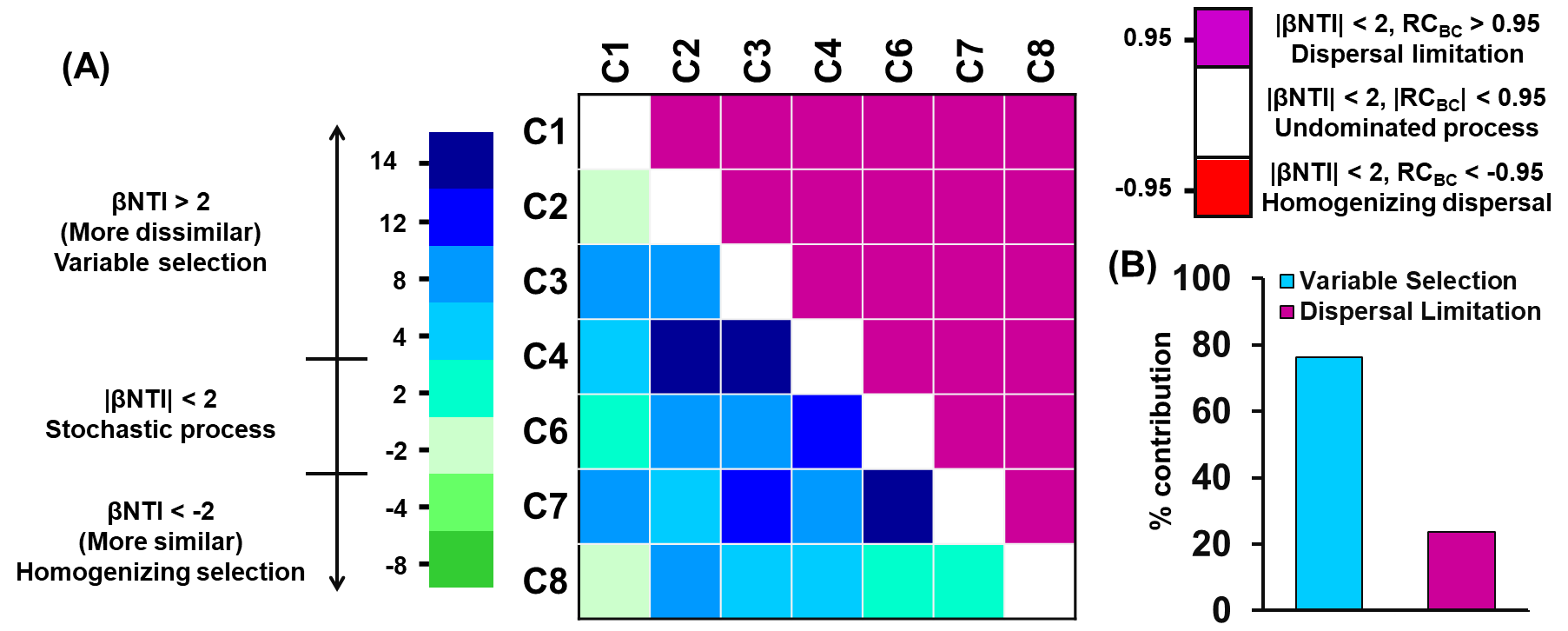


**Figure S10 (A)** Heatmap displaying β-nearest taxon index [βNTI (blue to green – lower triangle)] and Raup-Crick (Bray-Curtis) [RC_BC_ (pink to red - upper triangle)] between the microbial communities present within the Archaean granitic basement. Deterministic processes (left side of heatmap) include variable selection (blue; βNTI > 2) and homogenizing selection (green; βNTI < -2). When |βNTI| < 2, the phylogenetic relatedness between two communities did not differ significantly than expected by chance, and stochastic processes dominate (right side of heatmap). Stochastic processes include homogenizing dispersal (red; |βNTI| < 2 and RC_BC_ < -0.95), dispersal limitation and drift (purple; |βNTI| < 2 and RC_BC_ > 0.95), and undominated (|RC_BC_| < 0.95) processes. (**B**) Percentage contribution of ecological processes was mentioned as bar-plot.

**
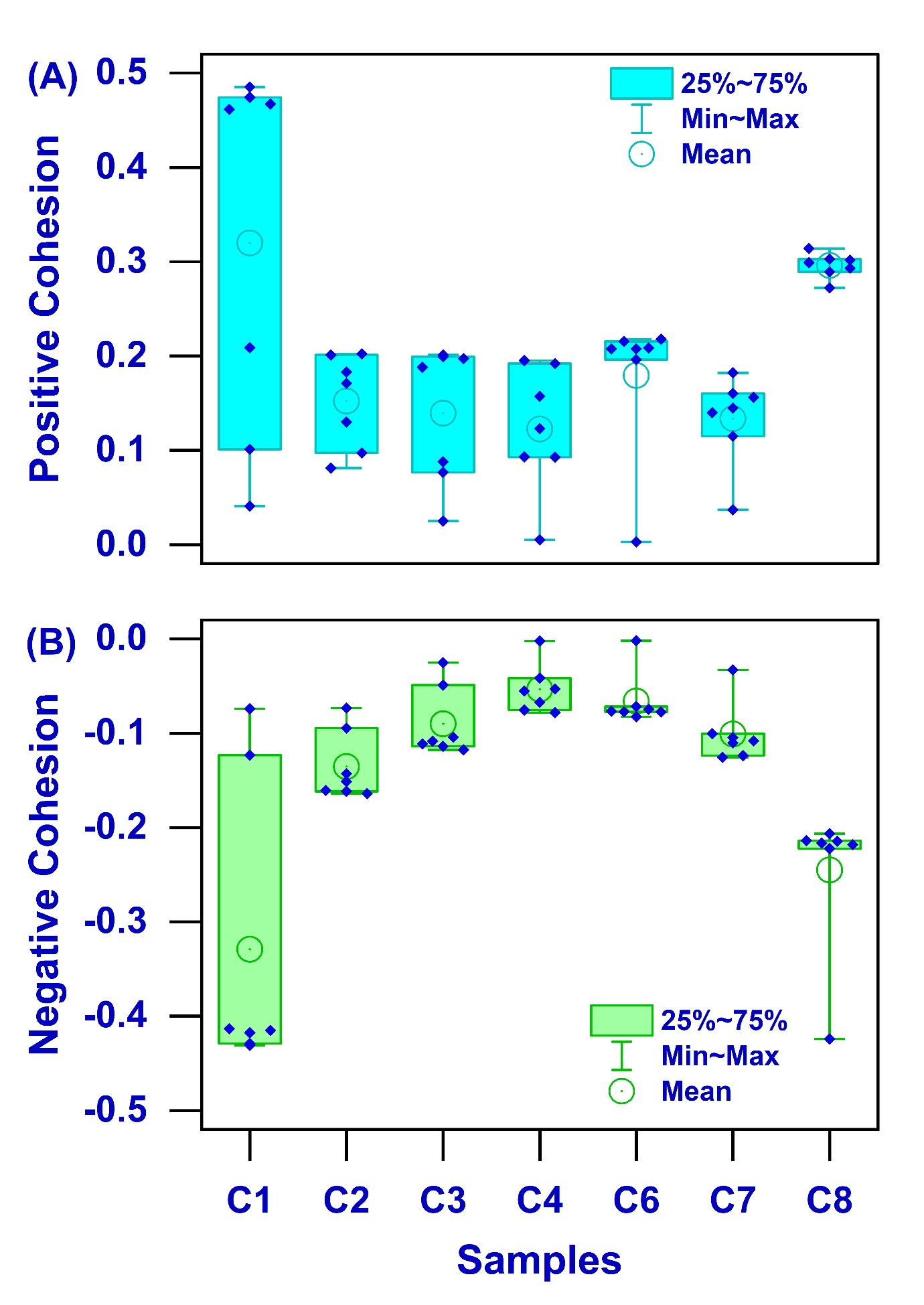
**

**Figure S11** Box-Whisker plot displaying **(A)** positive cohesion and **(B)** negative cohesion of the community of each horizon. Cohesion values were determined by considering all the ASVs with persistence cut-off > 0.5 in each sample.


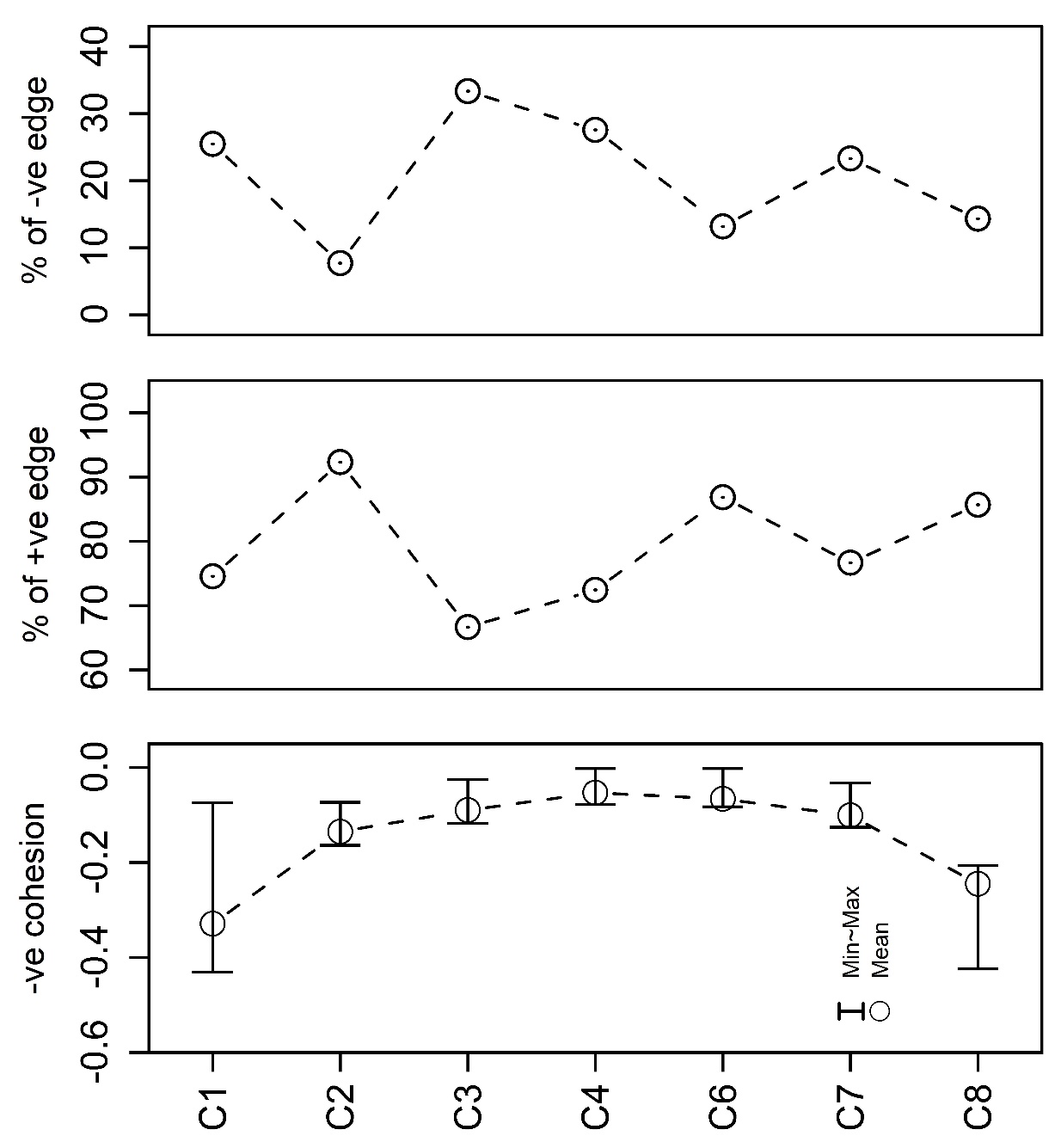


**Figure S12** Depth-wide pattern of –ve cohesion and network topologies (% of +ve and –ve edges).

**Table S1** Sequence read details and alpha diversity parameters of C1 subsamples

| **Samples Name** | **C1a** | **C1b** | **C1c** | **C1d** | **C1e** | **C1f** | **C1g** |
| --- | --- | --- | --- | --- | --- | --- | --- |
| Quality filtered reads | 204936 | 274371 | 382195 | 189064 | 442327 | 143447 | 136758 |
| No. of ASVs | 1497 | 1247 | 1276 | 1357 | 719 | 1333 | 1277 |
| No. of ASVs Bacteria | 1493 | 1244 | 1275 | 1355 | 713 | 1328 | 1273 |
| No. of ASVs Archaea | 4 | 3 | 1 | 2 | 6 | 5 | 4 |
| No. of ASVs Unassigned | 0 | 0 | 0 | 0 | 0 | 0 | 0 |
| % Abundance Bacteria | 99.94 | 99.98 | 99.96 | 99.95 | 99.84 | 99.92 | 99.93 |
| % Abundance Archaea | 0.06 | 0.02 | 0.04 | 0.05 | 0.16 | 0.08 | 0.07 |
| % Abundance Unassigned | 0 | 0 | 0 | 0 | 0 | 0 | 0 |
| No. of Phyla | 21 | 20 | 18 | 20 | 16 | 23 | 26 |
| No. of Class | 45 | 38 | 28 | 41 | 24 | 48 | 50 |
| No. of Family | 139 | 128 | 90 | 136 | 69 | 161 | 156 |
| No. of Genus | 220 | 188 | 123 | 189 | 68 | 226 | 216 |
| *Shannon | 7.1 | 6.41 | 8.41 | 7.14 | 8.35 | 7.37 | 7.3 |
| *Gini - Simpson | 0.97 | 0.97 | 0.99 | 0.97 | 0.99 | 0.98 | 0.98 |
| *Chao1 | 1493.07 | 1215.14 | 1259.6 | 1366.29 | 704 | 1334.84 | 1255.52 |
| *Goods coverage | 1 | 1 | 1 | 1 | 1 | 1 | 1 |
| *Faith’s PD index | 46.36 | 40.87 | 26.78 | 40.38 | 17.59 | 51.44 | 48.72 |
| *Observed ASVs | 1424 | 1107 | 1226 | 1295 | 695 | 1300 | 1230 |

* For alpha diversity analysis subsamples were normalised with 42000 reads

**Table S2** Sequence read details and alpha diversity parameters of C2 subsamples

| **Samples Name** | **C2a** | **C2b** | **C2c** | **C2d** | **C2e** | **C2f** | **C2g** |
| --- | --- | --- | --- | --- | --- | --- | --- |
| Quality filtered reads | 158018 | 172709 | 164927 | 166149 | 451469 | 46476 | 79488 |
| No. of ASVs | 1582 | 1537 | 1341 | 1527 | 832 | 988 | 1159 |
| No. of ASVs Bacteria | 1580 | 1534 | 1340 | 1514 | 820 | 982 | 1153 |
| No. of ASVs Archaea | 2 | 2 | 1 | 13 | 12 | 6 | 6 |
| No. of ASVs Unassigned | 0 | 1 | 0 | 0 | 0 | 0 | 0 |
| % Abundance Bacteria | 99.95 | 99.89 | 99.91 | 99.42 | 99.43 | 99.64 | 99.55 |
| % Abundance Archaea | 0.05 | 0.1 | 0.09 | 0.58 | 0.57 | 0.36 | 0.45 |
| % Abundance Unassigned | 0 | 0.02 | 0 | 0 | 0 | 0 | 0 |
| No. of Phyla | 20 | 17 | 21 | 19 | 16 | 24 | 22 |
| No. of Class | 41 | 34 | 34 | 31 | 26 | 41 | 41 |
| No. of Family | 125 | 123 | 117 | 121 | 74 | 134 | 134 |
| No. of Genus | 171 | 173 | 168 | 174 | 87 | 179 | 189 |
| *Shannon | 9.49 | 9.32 | 9.02 | 9.54 | 8.71 | 9.06 | 9.08 |
| *Gini - Simpson | 1 | 1 | 0.99 | 1 | 1 | 1 | 1 |
| *Chao1 | 1562.5 | 1534.54 | 1337.49 | 1516.67 | 818.67 | 987.46 | 1161.55 |
| *Goods coverage | 1 | 1 | 1 | 1 | 1 | 1 | 1 |
| *Faith’s PD index | 39.78 | 43.25 | 34.17 | 38.22 | 18.94 | 42.68 | 43.36 |
| *Observed ASVs | 1553 | 1509 | 1328 | 1501 | 810 | 987 | 1157 |

* For alpha diversity analysis subsamples were normalised with 42000 reads

**Table S3** Sequence read details and alpha diversity parameters of C3 subsamples

| **Samples Name** | **C3a** | **C3b** | **C3c** | **C3d** | **C3e** | **C3f** | **C3g** |
| --- | --- | --- | --- | --- | --- | --- | --- |
| Quality filtered reads | 155212 | 178731 | 205602 | 247883 | 419589 | 126911 | 123441 |
| No. of ASVs | 2150 | 2559 | 772 | 2661 | 2449 | 1663 | 1532 |
| No. of ASVs Bacteria | 2143 | 2557 | 770 | 2652 | 2449 | 1660 | 1531 |
| No. of ASVs Archaea | 7 | 2 | 2 | 8 | 0 | 3 | 1 |
| No. of ASVs Unassigned | 0 | 0 | 0 | 1 | 0 | 0 | 0 |
| % Abundance Bacteria | 99.86 | 99.98 | 99.99 | 99.88 | 100 | 99.85 | 99.89 |
| % Abundance Archaea | 0.14 | 0.02 | 0.01 | 0.12 | 0 | 0.15 | 0.11 |
| % Abundance Unassigned | 0 | 0 | 0 | 0 | 0 | 0 | 0 |
| No. of Phyla | 21 | 21 | 20 | 26 | 22 | 18 | 19 |
| No. of Class | 43 | 40 | 32 | 51 | 38 | 30 | 33 |
| No. of Family | 153 | 175 | 126 | 169 | 152 | 127 | 122 |
| No. of Genus | 233 | 286 | 169 | 269 | 230 | 198 | 189 |
| *Shannon | 9.58 | 10.53 | 5.82 | 10.51 | 10.05 | 10.05 | 9.84 |
| *Gini - Simpson | 0.99 | 1 | 0.96 | 1 | 1 | 1 | 1 |
| *Chao1 | 2131.43 | 2552.72 | 748.4 | 2650.48 | 2393.35 | 1666.74 | 1530.75 |
| *Goods coverage | 1 | 1 | 1 | 1 | 1 | 1 | 1 |
| *Faith’s PD index | 56.9 | 63.93 | 34.65 | 65.36 | 46.45 | 43.98 | 39.8 |
| *Observed ASVs | 2091 | 2515 | 694 | 2596 | 2359 | 1659 | 1525 |

* For alpha diversity analysis subsamples were normalised with 42000 reads

**Table S4** Sequence read details and alpha diversity parameters of C4 subsamples

| **Samples Name** | **C4a** | **C4b** | **C4c** | **C4d** | **C4e** | **C4f** | **C4g** |
| --- | --- | --- | --- | --- | --- | --- | --- |
| Quality filtered reads | 134911 | 135136 | 122761 | 160277 | 347937 | 120873 | 62777 |
| No. of ASVs | 1521 | 1243 | 449 | 1558 | 873 | 1040 | 936 |
| No. of ASVs Bacteria | 1521 | 1241 | 448 | 1547 | 873 | 1037 | 933 |
| No. of ASVs Archaea | 0 | 2 | 1 | 11 | 0 | 3 | 3 |
| No. of ASVs Unassigned | 0 | 0 | 0 | 0 | 0 | 0 | 0 |
| % Abundance Bacteria | 100 | 99.9 | 99.99 | 99.54 | 100 | 99.93 | 99.84 |
| % Abundance Archaea | 0 | 0.1 | 0.01 | 0.46 | 0 | 0.07 | 0.16 |
| % Abundance Unassigned | 0 | 0 | 0 | 0 | 0 | 0 | 0 |
| No. of Phyla | 16 | 20 | 19 | 22 | 11 | 15 | 18 |
| No. of Class | 36 | 32 | 27 | 46 | 17 | 26 | 33 |
| No. of Family | 154 | 99 | 102 | 142 | 70 | 86 | 109 |
| No. of Genus | 225 | 135 | 138 | 200 | 88 | 115 | 149 |
| *Shannon | 9.6 | 9.29 | 5.14 | 9.33 | 8.93 | 9.19 | 9.19 |
| *Gini - Simpson | 1 | 1 | 0.95 | 1 | 1 | 1 | 1 |
| *Chao1 | 1513.09 | 1245.8 | 447.09 | 1567.66 | 862.75 | 1039.14 | 937 |
| *Goods coverage | 1 | 1 | 1 | 1 | 1 | 1 | 1 |
| *Faith’s PD index | 47.52 | 31.32 | 27.06 | 45.63 | 18.04 | 28.00 | 37.75 |
| *Observed ASVs | 1495 | 1235 | 430 | 1531 | 850 | 1039 | 934 |

* For alpha diversity analysis subsamples were normalised with 42000 reads

**Table S5** Sequence read details and alpha diversity parameters of C6 subsamples

| **Samples Name** | **C6a** | **C6b** | **C6c** | **C6d** | **C6e** | **C6f** | **C6g** |
| --- | --- | --- | --- | --- | --- | --- | --- |
| Quality filtered reads | 122951 | 136703 | 158829 | 128793 | 117944 | 46733 | 71358 |
| No. of ASVs | 1451 | 1602 | 445 | 1579 | 1218 | 1023 | 1130 |
| No. of ASVs Bacteria | 1448 | 1594 | 444 | 1573 | 1217 | 1021 | 1126 |
| No. of ASVs Archaea | 3 | 8 | 1 | 6 | 1 | 2 | 4 |
| No. of ASVs Unassigned | 0 | 0 | 0 | 0 | 0 | 0 | 0 |
| % Abundance Bacteria | 99.87 | 99.79 | 100 | 99.71 | 99.96 | 99.79 | 99.87 |
| % Abundance Archaea | 0.13 | 0.21 | 0 | 0.29 | 0.04 | 0.21 | 0.13 |
| % Abundance Unassigned | 0 | 0 | 0 | 0 | 0 | 0 | 0 |
| No. of Phyla | 19 | 19 | 11 | 22 | 18 | 21 | 21 |
| No. of Class | 38 | 35 | 15 | 43 | 31 | 43 | 38 |
| No. of Family | 122 | 131 | 84 | 136 | 111 | 141 | 131 |
| No. of Genus | 169 | 186 | 111 | 198 | 141 | 193 | 189 |
| *Shannon | 9.29 | 9.57 | 5.16 | 9.53 | 9.33 | 9.22 | 9.25 |
| *Gini - Simpson | 1 | 1 | 0.95 | 1 | 1 | 1 | 1 |
| *Chao1 | 1453.65 | 1598.3 | 422.99 | 1573.76 | 1208.28 | 1021.27 | 1134.2 |
| *Goods coverage | 1 | 1 | 1 | 1 | 1 | 1 | 1 |
| *Faith’s PD index | 40.61 | 42.66 | 19.83 | 43.48 | 33.59 | 41.65 | 44.1 |
| *Observed ASVs | 1434 | 1590 | 377 | 1562 | 1205 | 1021 | 1124 |

* For alpha diversity analysis subsamples were normalised with 42000 reads

**Table S6** Sequence read details and alpha diversity parameters of C7 subsamples

| **Samples Name** | **C7a** | **C7b** | **C7c** | **C7d** | **C7e** | **C7f** | **C7g** |
| --- | --- | --- | --- | --- | --- | --- | --- |
| Quality filtered reads | 117269 | 107497 | 194815 | 106288 | 101837 | 84093 | 75411 |
| No. of ASVs | 1441 | 1247 | 1952 | 1305 | 1529 | 1242 | 1251 |
| No. of ASVs Bacteria | 1438 | 1243 | 1952 | 1295 | 1522 | 1237 | 1243 |
| No. of ASVs Archaea | 3 | 4 | 0 | 10 | 7 | 5 | 8 |
| No. of ASVs Unassigned | 0 | 0 | 0 | 0 | 0 | 0 | 0 |
| % Abundance Bacteria | 99.93 | 99.88 | 100 | 99.6 | 99.67 | 99.54 | 99.6 |
| % Abundance Archaea | 0.07 | 0.12 | 0 | 0.4 | 0.33 | 0.46 | 0.4 |
| % Abundance Unassigned | 0 | 0 | 0 | 0 | 0 | 0 | 0 |
| No. of Phyla | 22 | 24 | 20 | 20 | 22 | 22 | 25 |
| No. of Class | 38 | 41 | 48 | 37 | 40 | 43 | 44 |
| No. of Family | 136 | 123 | 206 | 123 | 144 | 134 | 147 |
| No. of Genus | 177 | 171 | 379 | 167 | 217 | 181 | 203 |
| *Shannon | 9.68 | 9.29 | 7.13 | 9.54 | 9.88 | 9.45 | 9.25 |
| *Gini - Simpson | 1 | 1 | 0.97 | 1 | 1 | 1 | 1 |
| *Chao1 | 1435.92 | 1249.33 | 1838.1 | 1304.23 | 1527.33 | 1241.2 | 1256.22 |
| *Goods coverage | 1 | 1 | 0.99 | 1 | 1 | 1 | 1 |
| *Faith’s PD index | 43.09 | 40.67 | 67.13 | 39.24 | 50.77 | 44.57 | 45.01 |
| *Observed ASVs | 1420 | 1241 | 1699 | 1297 | 1524 | 1239 | 1249 |

* For alpha diversity analysis subsamples were normalised with 42000 reads

**Table S7** Sequence read details and alpha diversity parameters of C8 subsamples

| **Samples Name** | **C8a** | **C8b** | **C8c** | **C8d** | **C8e** | **C8f** | **C8g** |
| --- | --- | --- | --- | --- | --- | --- | --- |
| Quality filtered reads | 116230 | 85324 | 109734 | 93265 | 176372 | 59471 | 61755 |
| No. of ASVs | 2195 | 1953 | 2234 | 2102 | 2604 | 1575 | 725 |
| No. of ASVs Bacteria | 2193 | 1949 | 2222 | 2100 | 2597 | 1573 | 721 |
| No. of ASVs Archaea | 2 | 4 | 12 | 2 | 7 | 2 | 4 |
| No. of ASVs Unassigned | 0 | 0 | 0 | 0 | 0 | 0 | 0 |
| % Abundance Bacteria | 99.96 | 99.94 | 99.65 | 99.98 | 99.85 | 99.96 | 99.92 |
| % Abundance Archaea | 0.04 | 0.06 | 0.35 | 0.02 | 0.15 | 0.04 | 0.08 |
| % Abundance Unassigned | 0 | 0 | 0 | 0 | 0 | 0 | 0 |
| No. of Phyla | 20 | 24 | 27 | 21 | 25 | 23 | 18 |
| No. of Class | 48 | 49 | 52 | 52 | 51 | 50 | 39 |
| No. of Family | 190 | 183 | 192 | 186 | 199 | 180 | 127 |
| No. of Genus | 325 | 304 | 318 | 310 | 322 | 279 | 158 |
| *Shannon | 10.09 | 10.01 | 10.14 | 10.06 | 10.15 | 9.84 | 5.48 |
| *Gini - Simpson | 1 | 1 | 1 | 1 | 1 | 1 | 0.93 |
| *Chao1 | 2193.33 | 1946.06 | 2229.18 | 2121.57 | 2558.56 | 1575.39 | 720.57 |
| *Goods coverage | 1 | 1 | 1 | 1 | 1 | 1 | 1 |
| *Faith’s PD index | 68.55 | 63.99 | 63.75 | 67.46 | 69.84 | 57.41 | 34.67 |
| *Observed ASVs | 2161 | 1932 | 2205 | 2074 | 2506 | 1571 | 716 |

* For alpha diversity analysis subsamples were normalised with 42000 reads

**Table S8** Permutational Analysis of variance (PERMANOVA) explaining the significant variability between the microbial communities.

| PERMANOVA | | | | | |
| --- | --- | --- | --- | --- | --- |
|  | **Df** | **Sum of Sqs** | **R^2^** | **F** | **Pr(>F)** |
| Model | 2 | 0.63 | 0.40 | 1.35 | 0.021 |
| Residual | 4 | 0.93 | 0.60 |  |  |
| Total | 6 | 1.55 | 1 |  |  |

**Table S9** List of microbial classes with mean relative abundance >1% and their relative abundance in all three zones (SZ: Shallow Zone, IZ: Intermediate Zone, DZ: Deeper Zone)

| **Class** | **SH** | **IH** | **DH** |
| --- | --- | --- | --- |
| Gammaproteobacteria | 41.981 | 23.710 | 28.098 |
| Actinobacteria | 26.941 | 31.090 | 27.752 |
| Alphaproteobacteria | 9.812 | 10.571 | 14.389 |
| Bacilli | 7.313 | 8.203 | 10.122 |
| Cyanobacteriia | 1.437 | 5.687 | 4.619 |
| Bacteroidia | 4.580 | 2.747 | 3.188 |
| Verrucomicrobiae | 0.179 | 7.934 | 2.251 |
| Clostridia | 3.143 | 2.710 | 2.805 |
| Deinococci | 0.427 | 1.329 | 1.581 |

**Table S10** List of microbial classes of abundance 0.01 - 1% and their relative abundance in all three zones (SZ: Shallow Zone, IZ: Intermediate Zone, DZ: Deeper Zone)

| **Class** | **SH** | **IH** | **DH** |
| --- | --- | --- | --- |
| Acidimicrobiia | 0.272 | 0.444 | 0.491 |
| Chloroflexia | 0.266 | 0.377 | 0.474 |
| Thermoleophilia | 0.113 | 0.420 | 0.555 |
| Acidobacteriae | 0.492 | 0.263 | 0.190 |
| Limnochordia | 0.001 | 0.928 | 0.001 |
| Polyangia | 0.011 | 0.477 | 0.194 |
| Rubrobacteria | 0.216 | 0.270 | 0.139 |
| Planctomycetes | 0.259 | 0.159 | 0.191 |
| Desulfuromonadia | 0.141 | 0.202 | 0.110 |
| ua Bacteria | 0.070 | 0.154 | 0.220 |
| Negativicutes | 0.253 | 0.107 | 0.077 |
| Holophagae | 0.107 | 0.118 | 0.193 |
| Gemmatimonadetes | 0.061 | 0.115 | 0.233 |
| WPS-2 | 0.263 | 0.049 | 0.017 |
| Phycisphaerae | 0.060 | 0.135 | 0.111 |
| Fusobacteriia | 0.161 | 0.097 | 0.037 |
| Anaerolineae | 0.024 | 0.143 | 0.119 |
| Babeliae | 0.083 | 0.053 | 0.140 |
| Blastocatellia | 0.017 | 0.070 | 0.182 |
| Bdellovibrionia | 0.017 | 0.096 | 0.155 |
| Myxococcia | 0.086 | 0.060 | 0.096 |
| Nitrososphaeria | 0.082 | 0.059 | 0.093 |
| Rhodothermia | 0.033 | 0.112 | 0.078 |
| Parcubacteria | 0.060 | 0.058 | 0.087 |
| Syntrophia | 0.131 | 0.045 | 0.022 |
| Vampirivibrionia | 0.157 | 0.019 | 0.017 |
| Chloroflexi KD4-96 | 0.036 | 0.046 | 0.079 |
| Longimicrobia | 0.050 | 0.061 | 0.038 |
| Oligoflexia | 0.025 | 0.051 | 0.072 |
| Vicinamibacteria | 0.008 | 0.063 | 0.070 |
| Planctomycetota OM190 | 0.006 | 0.063 | 0.059 |
| Spirochaetia | 0.067 | 0.035 | 0.025 |
| Coriobacteriia | 0.005 | 0.037 | 0.061 |
| Ignavibacteria | 0.057 | 0.041 | 0.000 |
| Kryptonia | 0.053 | 0.033 | 0.011 |
| Methanosarcinia | 0.071 | 0.005 | 0.020 |
| Ktedonobacteria | 0.044 | 0.038 | 0.011 |
| Chlamydiae | 0.028 | 0.032 | 0.016 |
| Symbiobacteriia | 0.019 | 0.024 | 0.029 |
| Fimbriimonadia | 0.008 | 0.039 | 0.021 |
| Methanobacteria | 0.040 | 0.004 | 0.018 |
| Chloroflexi AD3 | 0.000 | 0.058 | 0.002 |
| Coprothermobacteria | 0.006 | 0.017 | 0.037 |
| Armatimonadia | 0.004 | 0.013 | 0.041 |
| Planctomycetota 028H05-P-BN-P5 | 0.004 | 0.030 | 0.021 |
| Kapabacteria | 0.020 | 0.019 | 0.012 |
| Desulfovibrionia | 0.022 | 0.009 | 0.017 |
| Chloroflexi OLB14 | 0.003 | 0.016 | 0.026 |
| Desulfobulbia | 0.003 | 0.037 | 0.003 |
| Thermoanaerobaculia | 0.002 | 0.002 | 0.027 |

**Table S11** List of rare microbial classes (mean relative abundance < 0.01%) and their relative abundance in all three zones (SZ: Shallow Zone, IZ: Intermediate Zone, DZ: Deeper Zone)

| **Class** | **SH** | **IH** | **DH** |
| --- | --- | --- | --- |
| Omnitrophia | 0.024138542 | 0.002390132 | 0 |
| Sumerlaeia | 0 | 0.000430238 | 0.015613035 |
| Bacteroidota SJA-28 | 0.015390569 | 0 | 0 |
| Altiarchaeia | 0 | 0 | 0.010835949 |
| Cloacimonadia | 0.010127207 | 0 | 0 |
| NB1-j | 0 | 0.007957674 | 0.000569678 |
| ua Chloroflexi | 0.000253793 | 0.007067531 | 0 |
| ua Proteobacteria | 5.63985E-05 | 0.006953169 | 0 |
| Leptospirillia | 0 | 0 | 0.005269522 |
| ua Archaea | 0.000169195 | 0 | 0.004319051 |
| Armatimonadota DG-56 | 0 | 0 | 0.003987746 |
| Deferribacteres | 0.003187448 | 0.000338044 | 0 |
| Micrarchaeia | 0 | 0.003170816 | 0 |
| Patescibacteria ABY1 | 0 | 0.0027218 | 0 |
| Chloroflexi SHA-26 | 0 | 0.002638373 | 0 |
| Methanocellia | 0 | 0.00224473 | 0 |
| Entotheonellia | 0 | 0 | 0.001718689 |
| Syntrophobacteria | 0.001694592 | 0 | 0 |
| Halanaerobiia | 0 | 0 | 0.001495405 |
| Unassigned | 0.001331466 | 6.86168E-05 | 0 |
| Methanomethylicia | 0 | 0.001193089 | 0 |
| Desulfobacteria | 0.000930575 | 0 | 0 |
| Gracilibacteria | 0 | 0 | 0.000889216 |
| u Desulfobacterota | 0.000761379 | 0 | 0 |
| Zetaproteobacteria | 0 | 0 | 0.000712098 |
| MBNT15 | 0.000592184 | 0 | 0 |
| Syntrophorhabdia | 0 | 0 | 0.000569678 |
| BD2-11_terrestrial_group | 0.000479387 | 0 | 0 |
| ua Verrucomicrobiota | 0.000422988 | 0 | 0 |
| RCP2-54 | 0.000394789 | 0 | 0 |
| Pla4 lineage | 0 | 0.000343084 | 0 |
| Thermovenabulia | 0 | 0 | 0.000284839 |
| Acetothermiia | 0 | 0.00020585 | 0 |
| Microgenomatia | 0 | 0.000153656 | 0 |
| Sulfobacillia | 0 | 0 | 0.000127031 |
| Desulfomonilia | 0 | 0.000114361 | 0 |
| RBG-16-55-12 | 8.06949E-05 | 0 | 0 |
| Campylobacteria | 0.011188279 | 0.011733473 | 0.006659467 |
| Thermoplasmata | 0.009080318 | 0.007380508 | 0.010127473 |
| Chloroflexi JG30-KF-CM66 | 0.001917548 | 0.004240913 | 0.019758889 |
| ua Firmicutes | 0 | 0.017925506 | 0.007731086 |
| Chloroflexi Gitt-GS-136 | 0.006642541 | 0.007851201 | 0.010747089 |
| Thermoanaerobacteria | 0 | 0.011957788 | 0.012315665 |
| Halobacteria | 0.002312337 | 0.013314753 | 0.008476072 |
| Actinobacteriota MB-A2-108 | 0.001156168 | 0.009955339 | 0.01180573 |
| Desulfotomaculia | 0.002703278 | 0.006000171 | 0.013699727 |
| Latescibacterota | 0.001071571 | 0.001489404 | 0.017680229 |
| u Armatimonadota | 0.004579599 | 0.00833001 | 0.006457005 |
| Desulfitobacteriia | 0.001373957 | 0.01205309 | 0.005554361 |
| Moorellia | 0.005300686 | 0 | 0.012201912 |
| Saccharimonadia | 0.007537935 | 0.007318175 | 0.000996937 |
| SAR324_clade(Marine_group_B) | 0.002017372 | 0.012557731 | 0.000640888 |
| Methanomicrobia | 0.012386664 | 0.002711719 | 0 |
| Chloroflexi TK10 | 0.004737471 | 0 | 0.006813053 |
| Methylomirabilia | 0.004370881 | 0.003662811 | 0.003418068 |
| FCPU426 | 0.002098067 | 0.000450291 | 0.008345418 |
| Leptospirae | 0.005819981 | 0.004240913 | 0 |
| S0134 terrestrial group | 0 | 0.004121129 | 0.005908748 |
| Sericytochromatia | 0.004768804 | 0.004086789 | 0.000783307 |
| Aminicenantia | 0 | 0.007713545 | 0.001851454 |
| Dehalococcoidia | 0.001917548 | 0 | 0.006615113 |
| ua Acidobacteriota | 0 | 0.000799013 | 0.006420794 |
| Kiritimatiellae | 0 | 0.003718482 | 0.002858195 |
| Hydrogenedentia | 0.001063765 | 0.005283494 | 0 |
| Thermodesulfovibrionia | 0.001613898 | 0.000352352 | 0.004272585 |
| Elusimicrobia | 0.000967019 | 0.004456032 | 0 |
| Incertae_Sedis | 0 | 0.002468155 | 0.002032495 |
| Synergistia | 0 | 0.000731913 | 0.003429834 |
| GAL15 | 0.001353563 | 0 | 0.002604134 |
| Chthonomonadetes | 0.002095428 | 0 | 0.001709034 |
| Myxococcota bacteriap25 | 0.002537931 | 0 | 0.001210566 |
| Fibrobacteria | 0 | 0.000425544 | 0.0029196 |
| Nitrospiria | 0.001754014 | 0.000480318 | 0.00069867 |
| Abditibacteria | 0 | 0.0011713 | 0.001495405 |
| Thermotogae | 0.002057719 | 0.000365956 | 0 |
| Acidobacteriota Subgroup 18 | 0.000958774 | 0.00038299 | 0 |
| Defferrisomatia | 0 | 0.000399506 | 0.000569678 |

**Table S12** Symbols assigned against the microbial classes of mean relative abundance 0.01 - 1% in piper diagram Figure 3B(ii)

| **Class** |  |  | **Class** |  |
| --- | --- | --- | --- | --- |
| Desulfobulbia |  |  | Spirochaetia |  |
| Chlamydiae |  |  | Ignavibacteria |  |
| Desulfuromonadia |  |  | Fimbriimonadia |  |
| ua Bacteria |  |  | Vicinamibacteria |  |
| Polyangia |  |  | Coprothermobacteria |  |
| Phycisphaerae |  |  | Chloroflexi KD4-96 |  |
| Negativicutes |  |  | Fusobacteriia |  |
| Gemmatimonadetes |  |  | Syntrophia |  |
| Rubrobacteria |  |  | Blastocatellia |  |
| Planctomycetes |  |  | Babeliae |  |
| WPS-2 |  |  | Myxococcia |  |
| Acidimicrobiia |  |  | Planctomycetota OM190 |  |
| Limnochordia |  |  | Nitrososphaeria |  |
| Thermoleophilia |  |  | Anaerolineae |  |
| Acidobacteriae |  |  | Bdellovibrionia |  |
| Thermoanaerobaculia |  |  | Parcubacteria |  |
| Oligoflexia |  |  | Rhodothermia |  |
| Ktedonobacteria |  |  | Holophagae |  |
| Methanobacteria |  |  | Vampirivibrionia |  |
| Longimicrobia |  |  | Chloroflexia |  |
| Coriobacteriia |  |  | Planctomycetota 028H05-P-BN-P5 |  |
| Symbiobacteriia |  |  | Armatimonadia |  |
| Chloroflexi AD3 |  |  | Desulfovibrionia |  |
| Methanosarcinia |  |  | Chloroflexi OLB14 |  |
| Kryptonia |  |  | Kapabacteria |  |

**Table S13** Symbols assigned against the rare microbial classes (mean relative abundance < 0.01) in piper diagram Figure 3B(iii)

| **Class** |  |  | **Class** |  |
| --- | --- | --- | --- | --- |
| Desulfitobacteriia |  |  | Dehalococcoidia |  |
| Campylobacteria |  |  | Saccharimonadia |  |
| Leptospirillia |  |  | Hydrogenedentia |  |
| ua Archaea |  |  | Kiritimatiellae |  |
| NB1-j |  |  | GAL15 |  |
| Chloroflexi SHA-26 |  |  | Chthonomonadetes |  |
| Armatimonadota DG-56 |  |  | Synergistia |  |
| Micrarchaeia |  |  | Elusimicrobia |  |
| ua Chloroflexi |  |  | Incertae Sedis |  |
| ua Proteobacteria |  |  | Myxococcota bacteriap25 |  |
| Patescibacteria ABY1 |  |  | Thermodesulfovibrionia |  |
| Omnitrophia |  |  | Fibrobacteria |  |
| Cloacimonadia |  |  | ua Acidobacteriota |  |
| Bacteroidota SJA-28 |  |  | Thermotogae |  |
| Altiarchaeia |  |  | Abditibacteria |  |
| Moorellia |  |  | Defferrisomatia |  |
| ua Verrucomicrobiota |  |  | Acidobacteriota Subgroup 18 |  |
| RBG-16-55-12 |  |  | Nitrospiria |  |
| ua Firmicutes |  |  | Syntrophorhabdia |  |
| BD2-11 terrestrial group |  |  | Methanocellia |  |
| Acetothermiia |  |  | Zetaproteobacteria |  |
| Thermoplasmata |  |  | Halanaerobiia |  |
| Chloroflexi Gitt-GS-136 |  |  | Syntrophobacteria |  |
| Desulfomonilia |  |  | Methanomethylicia |  |
| Sulfobacillia |  |  | Pla4 lineage |  |
| Thermovenabulia |  |  | Desulfobacteria |  |
| Microgenomatia |  |  | Entotheonellia |  |
| Chloroflexi JG30-KF-CM66 |  |  | Unassigned |  |
| RCP2-54 |  |  | u Desulfobacterota |  |
| Thermoanaerobacteria |  |  | Gracilibacteria |  |
| Methanomicrobia |  |  | Deferribacteres |  |
| SAR324 clade (Marine group B) |  |  | MBNT15 |  |
| S0134 terrestrial group |  |  | Sumerlaeia |  |
| Sericytochromatia |  |  | Actinobacteriota MB-A2-108 |  |
| Leptospirae |  |  | Halobacteria |  |
| Methylomirabilia |  |  | Latescibacterota |  |
| FCPU426 |  |  | u Armatimonadota |  |
| Aminicenantia |  |  | Desulfotomaculia |  |
| Chloroflexi TK10 |  |  |  |  |

**Table S14** Similarity Percentage (SIMPER) analysis explaining dissimilarities between the distribution of microbial classes across three zones.

| **Microbial class categories** | **Horizons** | **Average dissimilarity** |
| --- | --- | --- |
| Microbial class  (abundance >1%) | SH and IH | 26.82 |
|  | IH and DH | 14.86 |
|  | SH and DH | 24.83 |
|  | SH, IH and DH | 21.84 |
| Microbial class with  (abundance 1.0 - 0.01%) | SH and IH | 52.78 |
|  | IH and DH | 40.49 |
|  | SH and DH | 53.84 |
|  | SH, IH and DH | 49.11 |
| Microbial class  (abundance < 0.01%) | SH and IH | 80.12 |
|  | IH and DH | 72.54 |
|  | SH and DH | 77.65 |
|  | SH, IH and DH | 76.77 |
| Total microbial class | SH and IH | 28.2 |
|  | IH and DH | 16.36 |
|  | SH and DH | 26.25 |
|  | SH, IH and DH | 23.28 |

**Table S15** List of major microbial genera (mean relative abundance > 0.5%) and their abundance in three zones.

| **Domain; Phylum; Class; Order; Family; Genus** | **SH** | **IH** | **DH** |
| --- | --- | --- | --- |
| Bacteria; Actinobacteriota; Actinobacteria; Corynebacteriales; Corynebacteriaceae; Corynebacterium | 12.77 | 10.02 | 6.33 |
| Bacteria; Actinobacteriota; Actinobacteria; Micrococcales; Micrococcaceae; Micrococcus | 2.92 | 5.58 | 4.32 |
| Bacteria; Actinobacteriota; Actinobacteria; Propionibacteriales; Nocardioidaceae; Nocardioides | 0.31 | 1.16 | 2.69 |
| Bacteria; Actinobacteriota; Actinobacteria; Pseudonocardiales; Pseudonocardiaceae; Pseudonocardia | 1.34 | 2.04 | 0.74 |
| Bacteria; Actinobacteriota; Actinobacteria; Micrococcales; Micrococcaceae; Kocuria | 0.9 | 1.9 | 1.14 |
| Bacteria; Actinobacteriota; Actinobacteria; Corynebacteriales; Corynebacteriaceae; Lawsonella | 1.75 | 0.78 | 0.58 |
| Bacteria; Actinobacteriota; Actinobacteria; Micrococcales; Intrasporangiaceae | 1.06 | 1.01 | 0.8 |
| Bacteria; Actinobacteriota; Actinobacteria; Micrococcales; Micrococcaceae | 0.67 | 0.32 | 1.82 |
| Bacteria; Actinobacteriota; Actinobacteria; Corynebacteriales; Dietziaceae; Dietzia | 0.09 | 0.11 | 2.12 |
| Bacteria; Actinobacteriota; Actinobacteria; Micrococcales; Dermabacteraceae; Brachybacterium | 0.41 | 0.63 | 0.75 |
| Bacteria; Actinobacteriota; Actinobacteria; Micrococcales; Brevibacteriaceae; Brevibacterium | 0.66 | 0.72 | 0.31 |
| Bacteria; Proteobacteria; Gammaproteobacteria; Pseudomonadales; Pseudomonadaceae; Pseudomonas | 14.95 | 8.94 | 9.45 |
| Bacteria; Proteobacteria; Gammaproteobacteria; Pseudomonadales; Moraxellaceae; Acinetobacter | 3.69 | 3.67 | 3.86 |
| Bacteria; Proteobacteria; Gammaproteobacteria; Xanthomonadales; Xanthomonadaceae; Stenotrophomonas | 8.8 | 1.07 | 1.27 |
| Bacteria; Proteobacteria; Gammaproteobacteria; Burkholderiales; Comamonadaceae | 2.88 | 1.06 | 1.07 |
| Bacteria; Proteobacteria; Gammaproteobacteria; Burkholderiales; Comamonadaceae; Comamonas | 0.2 | 0.26 | 4.19 |
| Bacteria; Proteobacteria; Gammaproteobacteria; Burkholderiales; Oxalobacteraceae; Massilia | 2.3 | 0.21 | 0.26 |
| Bacteria; Proteobacteria; Gammaproteobacteria; Burkholderiales; Burkholderiaceae; Ralstonia | 2.11 | 0.17 | 0.44 |
| Bacteria; Proteobacteria; Gammaproteobacteria; Xanthomonadales; Xanthomonadaceae; Pseudoxanthomonas | 0.73 | 1.23 | 0.75 |
| Bacteria; Proteobacteria; Gammaproteobacteria; Pseudomonadales; Moraxellaceae; Enhydrobacter | 0.48 | 0.84 | 1.35 |
| Bacteria; Proteobacteria; Gammaproteobacteria; Alteromonadales; Shewanellaceae; Shewanella | 0.07 | 1.18 | 0.75 |
| Bacteria; Proteobacteria; Alphaproteobacteria; Rhodobacterales; Rhodobacteraceae; Paracoccus | 1.22 | 1.66 | 1.66 |
| Bacteria; Proteobacteria; Alphaproteobacteria; Caulobacterales; Caulobacteraceae; Brevundimonas | 0.76 | 0.42 | 2.07 |
| Bacteria; Proteobacteria; Alphaproteobacteria; Sphingomonadales; Sphingomonadaceae; Sphingomonas | 0.83 | 1.01 | 1.34 |
| Bacteria; Proteobacteria; Alphaproteobacteria; Rhizobiales; Rhizobiaceae; Allorhizobium-Neorhizobium-Pararhizobium-Rhizobium | 1.06 | 1 | 0.91 |
| Bacteria; Proteobacteria; Alphaproteobacteria; Rhizobiales; Beijerinckiaceae; Methylobacterium-Methylorubrum | 1.25 | 0.85 | 0.71 |
| Bacteria; Proteobacteria; Alphaproteobacteria; Acetobacterales; Acetobacteraceae; Craurococcus-Caldovatus | 0.11 | 0.18 | 1.74 |
| Bacteria; Firmicutes; Bacilli; Bacillales; Bacillaceae; Bacillus | 1.88 | 1.49 | 2.29 |
| Bacteria; Firmicutes; Bacilli; Erysipelotrichales; Erysipelotrichaceae; Erysipelothrix | 1.18 | 1.53 | 0.3 |
| Bacteria; Firmicutes; Bacilli; Exiguobacterales; Exiguobacteraceae; Exiguobacterium | 1.09 | 0.71 | 0.69 |
| Bacteria; Firmicutes; Bacilli; Lactobacillales; Carnobacteriaceae | 0.04 | 0.1 | 2.06 |
| Bacteria; Firmicutes; Bacilli; Bacillales; Planococcaceae; Lysinibacillus | 0.92 | 0.23 | 0.89 |
| Bacteria; Firmicutes; Clostridia; Peptostreptococcales-Tissierellales; Peptostreptococcales-Tissierellales; Anaerococcus | 0.94 | 0.68 | 0.49 |
| Bacteria; Verrucomicrobiota; Verrucomicrobiae; Opitutales; Opitutaceae; IMCC26134 | 0.09 | 7.76 | 2.12 |
| Bacteria; Cyanobacteria; Cyanobacteriia; Chloroplast; Chloroplast; Chloroplast | 1.32 | 1.87 | 2.03 |
| Bacteria; Cyanobacteria; Cyanobacteriia; Cyanobacteriales | 0 | 1.62 | 0.14 |
| Bacteria; Deinococcota; Deinococci; Deinococcales; Deinococcaceae; Deinococcus | 0.36 | 0.88 | 1.18 |

**Table 16** List of genera in each clique

| **Sl. no** | **Clique no.** | **Genera** |
| --- | --- | --- |
| 1 | 1 | Micrococcus |
| 2 | 1 | Opitutaceae IMCC26134 |
| 3 | 1 | Chloroplast |
| 4 | 1 | Pseudoxanthomonas |
| 5 | 2 | Acinetobacter |
| 6 | 2 | Paracoccus |
| 7 | 2 | Shewanella |
| 8 | 3 | Corynebacterium |
| 9 | 3 | ua Comamonadaceae |
| 10 | 3 | Lawsonella |
| 11 | 3 | Allorhizobium-Neorhizobium-Pararhizobium-Rhizobium (ANPR) |
| 12 | 3 | Methylobacterium-Methylorubrum |
| 13 | 3 | Anaerococcus |
| 14 | 4 | Bacillus |
| 15 | 4 | Sphingomonas |
| 16 | 4 | ua Intrasporangiaceae |
| 17 | 4 | Exiguobacterium |
| 18 | 4 | Dietzia |
| 19 | 4 | Craurococcus-Caldovatus |
| 20 | 4 | Brachybacterium |
| 21 | 5 | Pseudonocardia |
| 22 | 5 | Erysipelothrix |
| 23 | 6 | Nocardioides |
| 24 | 6 | Enhydrobacter |
| 25 | 6 | Deinococcus |
| 26 | 6 | ua Cyanobacteriales |
| 27 | 6 | ua Carnobacteriaceae |
| 28 | 7 | Brevundimonas |
| 29 | 7 | Lysinibacillus |
| 30 | 8 | Pseudomonas |
| 31 | 8 | Stenotrophomonas |

**Table S17** Detrended Correspondence Analysis (DCA) of microbial communities

|  | **DCA1** | **DCA2** | **DCA3** | **DCA4** |
| --- | --- | --- | --- | --- |
| **Eigenvalues** | 0.126 | 0.0269 | 0.027345 | 0.027857 |
| **Additive Eigenvalues** | 0.126 | 0.02524 | 0.031761 | 0.002497 |
| **Decorana values** | 0.1261 | 0.02034 | 0.003892 | 0.000733 |
| **Axis lengths** | 0.9032 | 0.4608 | 0.475491 | 0.470558 |

**Table S18** Pairwise spearman correlation among the geochemical parameters

|  | **Depth** | **Temp** | **TIC** | **TOC** | **Mn** | **Fe** | **Fe_2_O_3_** | **NO_2_^-^** | **Cl^-^** | **NO_3_^-^** | **SO_4_^2-^** | **PO_4_^3-^** | **CH_4_** | **CO_2_** | **H_2_** | **He** |
| --- | --- | --- | --- | --- | --- | --- | --- | --- | --- | --- | --- | --- | --- | --- | --- | --- |
| **Depth** |  | 0.99 | 0.14 | 0.61 | 0.89 | -0.64 | -0.07 | -0.76 | -0.64 | -0.82 | -0.86 | -0.89 | 0.77 | -0.20 | 0.62 | 0.67 |
| **Temp** | 0.99 |  | 0.04 | 0.65 | 0.88 | -0.63 | -0.04 | -0.70 | -0.67 | -0.81 | -0.88 | -0.88 | 0.78 | -0.20 | 0.62 | 0.67 |
| **TIC** | 0.14 | 0.04 |  | 0.04 | -0.11 | 0.21 | 0.04 | -0.41 | 0.29 | -0.04 | 0.07 | 0.11 | 0.11 | -0.43 | -0.25 | -0.07 |
| **TOC** | 0.61 | 0.65 | 0.04 |  | 0.39 | -0.14 | 0.04 | -0.52 | -0.54 | -0.61 | -0.43 | -0.39 | 0.25 | -0.54 | -0.11 | 0.40 |
| **Mn** | 0.89 | 0.88 | -0.11 | 0.39 |  | -0.89 | -0.39 | -0.61 | -0.64 | -0.86 | -0.71 | -1 | 0.59 | 0.02 | 0.78 | 0.56 |
| **Fe** | -0.64 | -0.63 | 0.21 | -0.14 | -0.89 |  | 0.68 | 0.27 | 0.32 | 0.82 | 0.50 | 0.89 | -0.41 | -0.13 | -0.80 | -0.16 |
| **Fe_2_O_3_** | -0.07 | -0.04 | 0.04 | 0.04 | -0.39 | 0.68 |  | -0.09 | -0.14 | 0.46 | -0.25 | 0.39 | 0.29 | 0.18 | -0.29 | 0.40 |
| **NO_2_^-^** | -0.76 | -0.70 | -0.41 | -0.52 | -0.61 | 0.27 | -0.09 |  | 0.74 | 0.54 | 0.49 | 0.61 | -0.39 | 0.10 | -0.15 | -0.79 |
| **Cl^-^** | -0.64 | -0.67 | 0.29 | -0.54 | -0.64 | 0.32 | -0.14 | 0.74 |  | 0.43 | 0.50 | 0.64 | -0.27 | -0.13 | -0.27 | -0.92 |
| **NO_3_^-^** | -0.82 | -0.81 | -0.04 | -0.61 | -0.86 | 0.82 | 0.46 | 0.54 | 0.43 |  | 0.64 | 0.86 | -0.52 | 0.13 | -0.49 | -0.27 |
| **SO_4_^2-^** | -0.86 | -0.88 | 0.07 | -0.43 | -0.71 | 0.50 | -0.25 | 0.49 | 0.50 | 0.64 |  | 0.71 | -0.95 | -0.09 | -0.65 | -0.59 |
| **PO_4_^3-^** | -0.89 | -0.88 | 0.11 | -0.39 | -1.00 | 0.89 | 0.39 | 0.61 | 0.64 | 0.86 | 0.71 |  | -0.59 | -0.02 | -0.78 | -0.56 |
| **CH_4_** | 0.77 | 0.78 | 0.11 | 0.25 | 0.59 | -0.41 | 0.29 | -0.39 | -0.27 | -0.52 | -0.95 | -0.59 |  | 0.05 | 0.66 | 0.45 |
| **CO_2_** | -0.20 | -0.20 | -0.43 | -0.54 | 0.02 | -0.13 | 0.18 | 0.10 | -0.13 | 0.13 | -0.09 | -0.02 | 0.05 |  | 0.18 | 0.13 |
| **H_2_** | 0.62 | 0.62 | -0.25 | -0.11 | 0.78 | -0.80 | -0.29 | -0.15 | -0.27 | -0.49 | -0.65 | -0.78 | 0.66 | 0.18 |  | 0.31 |
| **He** | 0.67 | 0.67 | -0.07 | 0.40 | 0.56 | -0.16 | 0.40 | -0.79 | -0.92 | -0.27 | -0.59 | -0.56 | 0.45 | 0.13 | 0.31 |  |

**Table S19** Explainability of each independent geochemical variable

|  | **R^2^** | **Adj. R^2^** |
| --- | --- | --- |
| **Depth** | 0.63 | 0.55 |
| **TIC** | 0.04 | -0.15 |
| **TOC** | 0.39 | 0.27 |
| **NO_2_^-^** | 0.65 | 0.59 |
| **Fe_2_O_3_** | 0.19 | 0.03 |
| **CO_2_** | 0.06 | -0.13 |

**Table S20** Relative importance of each ecological process in different samples. HeS: Heterogeneous selection, HoS: Homogeneous selection, DL: Dispersal limitation, HD: Homogenizing dispersal, DR: Drift and others.

| **Method** | **Sample 1** | **Sample 2** | **HeS** | **HoS** | **DL** | **HD** | **DR** |
| --- | --- | --- | --- | --- | --- | --- | --- |
| bNRIaRCbraya | C1 | C2 | 0.47 | 0 | 0.30 | 0.05 | 0.17 |
| bNRIaRCbraya | C1 | C3 | 0.32 | 0 | 0.47 | 0.07 | 0.14 |
| bNRIaRCbraya | C1 | C4 | 0.43 | 0 | 0.35 | 0.03 | 0.19 |
| bNRIaRCbraya | C1 | C6 | 0.38 | 0 | 0.40 | 0.03 | 0.19 |
| bNRIaRCbraya | C1 | C7 | 0.47 | 0 | 0.32 | 0.02 | 0.19 |
| bNRIaRCbraya | C1 | C8 | 0.33 | 0 | 0.50 | 0.05 | 0.12 |
| bNRIaRCbraya | C2 | C3 | 0.37 | 0 | 0.43 | 0.03 | 0.17 |
| bNRIaRCbraya | C2 | C4 | 0.38 | 0 | 0.39 | 0.02 | 0.21 |
| bNRIaRCbraya | C2 | C6 | 0.34 | 0 | 0.42 | 0.03 | 0.20 |
| bNRIaRCbraya | C2 | C7 | 0.38 | 0 | 0.40 | 0.02 | 0.20 |
| bNRIaRCbraya | C2 | C8 | 0.34 | 0 | 0.48 | 0.02 | 0.17 |
| bNRIaRCbraya | C3 | C4 | 0.34 | 0 | 0.47 | 0.05 | 0.14 |
| bNRIaRCbraya | C3 | C6 | 0.38 | 0 | 0.42 | 0.07 | 0.13 |
| bNRIaRCbraya | C3 | C7 | 0.35 | 0 | 0.45 | 0.04 | 0.16 |
| bNRIaRCbraya | C3 | C8 | 0.25 | 0 | 0.55 | 0.01 | 0.19 |
| bNRIaRCbraya | C4 | C6 | 0.39 | 0 | 0.34 | 0.03 | 0.24 |
| bNRIaRCbraya | C4 | C7 | 0.37 | 0 | 0.40 | 0.03 | 0.20 |
| bNRIaRCbraya | C4 | C8 | 0.32 | 0 | 0.49 | 0.03 | 0.16 |
| bNRIaRCbraya | C6 | C7 | 0.42 | 0 | 0.32 | 0.05 | 0.21 |
| bNRIaRCbraya | C6 | C8 | 0.29 | 0 | 0.46 | 0.05 | 0.19 |
| bNRIaRCbraya | C7 | C8 | 0.34 | 0 | 0.45 | 0.02 | 0.19 |

**Table S21** Detailed topology of each network

| **Network Indices** | **C1** | **C2** | **C3** | **C4** | **C6** | **C7** | **C8** |
| --- | --- | --- | --- | --- | --- | --- | --- |
| Total nodes | 452 | 301 | 688 | 195 | 348 | 266 | 856 |
| Total links | 1139 | 702 | 1473 | 272 | 623 | 253 | 4001 |
| R square of power-law | 0.834 | 0.876 | 0.808 | 0.864 | 0.74 | 0.865 | 0.739 |
| Edge : Node | 2.52 | 2.33 | 2.14 | 1.39 | 1.79 | 0.95 | 4.67 |
| +ve edge | 849 | 648 | 982 | 197 | 541 | 194 | 3428 |
| -ve edge | 290 | 54 | 491 | 75 | 82 | 59 | 573 |
| % +ve edge | 74.54 | 92.31 | 66.67 | 72.43 | 86.84 | 76.68 | 85.68 |
| % -ve edge | 25.46 | 7.69 | 33.33 | 27.57 | 13.16 | 23.32 | 14.32 |
| Average degree (avgK) | 5.04 | 4.664 | 4.282 | 2.79 | 3.58 | 1.902 | 9.348 |
| Average clustering coefficient (avgCC) | 0.297 | 0.297 | 0.29 | 0.251 | 0.273 | 0.101 | 0.262 |
| Average path distance (GD) | 4.477 | 3.354 | 5.868 | 2.979 | 3.473 | 3.397 | 3.871 |
| Geodesic efficiency (E) | 0.272 | 0.382 | 0.228 | 0.449 | 0.339 | 0.394 | 0.31 |
| Harmonic geodesic distance (HD) | 3.675 | 2.618 | 4.394 | 2.226 | 2.951 | 2.539 | 3.229 |
| Maximal degree | 106 | 57 | 118 | 23 | 89 | 22 | 253 |
| Nodes with max degree | asv14100 | asv14098 | asv16238 | asv4506 | asv16238 | asv10483 | asv21812 |
| Centralization of degree (CD) | 0.225 | 0.176 | 0.166 | 0.105 | 0.248 | 0.076 | 0.286 |
| Maximal betweenness | 31179.951 | 2704.646 | 69496.838 | 350.167 | 10411.919 | 1172.75 | 39860.331 |
| Nodes with max betweenness | asv14100 | asv13471 | asv16238 | asv1200 | asv16238 | asv10483 | asv15737 |
| Centralization of betweenness (CB) | 0.303 | 0.059 | 0.291 | 0.018 | 0.171 | 0.033 | 0.107 |
| Maximal stress centrality | 518318 | 17830 | 4094171 | 1128 | 37044 | 4921 | 1048734 |
| Nodes with max stress centrality | asv14100 | asv13471 | asv16238 | asv4506 | asv16238 | asv4793 | asv2238 |
| Centralization of stress centrality (CS) | 5.012 | 0.389 | 17.073 | 0.057 | 0.604 | 0.138 | 2.811 |
| Maximal eigenvector centrality | 0.388 | 0.315 | 0.528 | 0.497 | 0.404 | 0.405 | 0.242 |
| Nodes with max eigenvector centrality | asv14100 | asv14098 | asv16238 | asv4506 | asv14104 | asv10483 | asv21812 |
| Centralization of eigenvector centrality (CE) | 0.369 | 0.292 | 0.513 | 0.475 | 0.381 | 0.386 | 0.226 |
| Density (D) | 0.011 | 0.016 | 0.006 | 0.014 | 0.01 | 0.007 | 0.011 |
| Reciprocity | 1 | 1 | 1 | 1 | 1 | 1 | 1 |
| Transitivity (Trans) | 0.171 | 0.21 | 0.087 | 0.228 | 0.109 | 0.132 | 0.087 |
| Connectedness (Con) | 0.701 | 0.211 | 0.667 | 0.101 | 0.393 | 0.065 | 0.776 |
| Efficiency | 0.987 | 0.939 | 0.993 | 0.896 | 0.98 | 0.93 | 0.987 |
| Hierarchy | 0 | 0 | 0 | 0 | 0 | 0 | 0 |
| Lubness | 1 | 1 | 1 | 1 | 1 | 1 | 1 |

**Table S22** Modularity of each network

| **Methods** | **Parameters** | **C1** | **C2** | **C3** | **C4** | **C6** | **C7** | **C8** |
| --- | --- | --- | --- | --- | --- | --- | --- | --- |
| Greedy modularity optimization | No. of modules | 33 | 46 | 44 | 30 | 50 | 71 | 50 |
|  | Modularity | 0.735 | 0.604 | 0.815 | 0.847 | 0.657 | 0.89 | 0.365 |
| Short random walks | No. of modules | 46 | 52 | 60 | 33 | 62 | 72 | 84 |
|  | Modularity | 0.721 | 0.597 | 0.814 | 0.838 | 0.633 | 0.886 | 0.335 |
| Leading eigenvector of the community matrix | No. of modules | 37 | 46 | 51 | 30 | 53 | 73 | 39 |
|  | Modularity | 0.712 | 0.594 | 0.731 | 0.847 | 0.607 | 0.889 | 0.23 |

**Table S23** Concentration of acetate measured from formation fluid during drilling of KFD1 and its depth-wide pattern

| **Depth (mbs)** | **CH_3_COO^-^ (mg/L) (±10%)** |
| --- | --- |
| 1833.0 | 195.41 |
| 1929.7 | 220.84 |
| 2025.9 | 224.33 |
| 2121.0 | 1207.57 |
| 2217.3 | 221.30 |
| 2313.0 | 975.75 |
| 2409.1 | 912.69 |
| 2514.0 | 753.47 |
| 2610.0 | 844.87 |
| 2763.0 | 959.79 |
| 2831.0 | 746.14 |

**References**

1. Roy, S. Scientific drilling in Koyna region, Maharashtra. *Curr. Sci.* **112**, 2181 (2017).

2. Goswami, D., Roy, S. & Akkiraju, V. V. Delineation of Damage Zones From 3 km Downhole Geophysical Logs in the Koyna Seismogenic Zone, Western India. *J. Geophys. Res. Solid Earth* **124**, 6101–6120 (2019).

3. Podugu, N., Mishra, S., Wiersberg, T. & Roy, S. Chemical and noble gas isotope compositions of formation gases from a 3 km deep scientific borehole in the Koyna Seismogenic Zone, Western India. *Geofluids* **2019**, (2019).

4. Kieft, T. L., Phelps, T. J. & Fredrickson, J. K. Drilling, coring, and sampling subsurface environments. *Man. Environ. Microbiol.* 799–817 (2007).

5. Colwell, F. S. *et al.* Estimates of biogenic methane production rates in deep marine sediments at Hydrate Ridge, Cascadia margin. *Appl. Environ. Microbiol.* **74**, 3444–3452 (2008).

6. Nyyssönen, M. *et al.* Taxonomically and functionally diverse microbial communities in deep crystalline rocks of the Fennoscandian shield. *ISME J.* **8**, 126–138 (2014).

7. Sahu, R. P. *et al.* Microbial diversity and function in crystalline basement beneath the Deccan Traps explored in a 3 km borehole at Koyna, western India. *Environ. Microbiol.* **24**, 2837–2853 (2022).

8. Chen, Y. *et al.* Microbial community dynamics and assembly mechanisms across different stages of cyanobacterial bloom in a large freshwater lake. *Sci. Total Environ.* **907**, 168207 (2024).

9. Zhu, H., Xiong, X., Liu, B. & Liu, G. Lakes-scale pattern of eukaryotic phytoplankton diversity and assembly process shaped by electrical conductivity in central Qinghai-Tibet Plateau. *FEMS Microbiol. Ecol.* **100**, 1–9 (2024).

10. Kieft, T. L. Microbiology of the Deep Continental Biosphere. 225–249 (2016) doi:10.1007/978-3-319-28071-4_6.

11. Kazy, S. K. *et al.* Microbial life in deep terrestrial continental crust. *Extrem. Environ.* 263–291 (2021).

12. Nuppunen-Puputti, M., Kietäväinen, R., Kukkonen, I. & Bomberg, M. Implications of a short carbon pulse on biofilm formation on mica schist in microcosms with deep crystalline bedrock groundwater. *Front. Microbiol.* **14**, 1–17 (2023).
